# Quantitative CT analysis in interstitial pneumonia with autoimmune features: diagnostic and prognostic insights from a retrospective cohort study

**DOI:** 10.1186/s13244-026-02253-0

**Published:** 2026-03-18

**Authors:** Marijan Pušeljić, Anselm Johannes Schlemmer, Igor Vlasicek, Ann-Katrin Kaufmann-Bühler, Florentine Moazedi-Fürst, Michael Fuchsjäger, Emina Talakić

**Affiliations:** 1 Division of General Radiology, Department of Radiology, Medical University of Graz, Graz, Austria; 2 Division of Rheumatology and Immunology, Department of Internal Medicine, Medical University of Graz, Graz, Austria

**Keywords:** Interstitial lung disease, Pulmonary fibrosis, Computed tomography, Quantitative lung analysis

## Abstract

**Objective:**

The position of interstitial pneumonia with autoimmune features (IPAF) within the interstitial lung disease (ILD) spectrum remains unclear, with limited data regarding quantitative CT (QCT). This study aims to evaluate threshold-based QCT for distinguishing IPAF from other ILD subtypes and to assess the prognostic value of specific QCT features.

**Materials and methods:**

In this retrospective single-centre study, 227 patients (mean age 63.6 ± 12.8 years) with CTD-ILD (*n* = 123), IPAF (*n* = 54), or IPF (*n* = 50) diagnosed between January 2005 and October 2024 were included. QCT assessed ground-glass opacity (GGO), consolidation, emphysema, affected lung, and GGO-to-consolidation ratio. Group comparisons used adjusted general linear models; progression-free survival (PFS) was analyzed with Kaplan–Meier and Cox regression to identify QCT-based risk factors.

**Results:**

Lung consolidation was significantly higher in IPAF than in CTD-ILD (*p* = 0.046), while CTD-ILD showed higher GGO-to-consolidation ratios than IPAF (*p* < 0.001) and IPF (*p* = 0.009). IPAF had shorter PFS than CTD-ILD but longer than IPF. Higher GGO-to-consolidation ratios (HR 0.87, 95% CI: 0.79–0.97, *p* = 0.011) and higher emphysema percentage (HR 0.96, 95% CI: 0.93–0.99, *p* = 0.011) were associated with reduced progression risk, whereas the usual interstitial pneumonia pattern with higher risk in some lung compartments (e.g., lower third, HR 1.70, 95% CI: 1.07–2.71, *p* = 0.024). In the exploratory subgroup analysis, the GGO-to-consolidation ratio was associated with lower PFS in CTD-ILD only.

**Conclusion:**

IPAF more closely resembled IPF in QCT features. The GGO-to-consolidation ratio emerged as a potential discriminative and prognostic factor.

**Critical relevance statement:**

Threshold-based QCT provides reproducible diagnostic and prognostic biomarkers that help distinguish IPAF from other ILD subtypes and support risk stratification.

**Key Points:**

- Quantitative CT (QCT) has not been systematically investigated in interstitial pneumonia with autoimmune features (IPAF).
- QCT revealed distinct imaging and prognostic differences when comparing IPAF with other interstitial lung disease subtypes.
- QCT provides reproducible imaging biomarkers that aid IPAF differentiation and support clinical risk stratification.

**Graphical Abstract:**

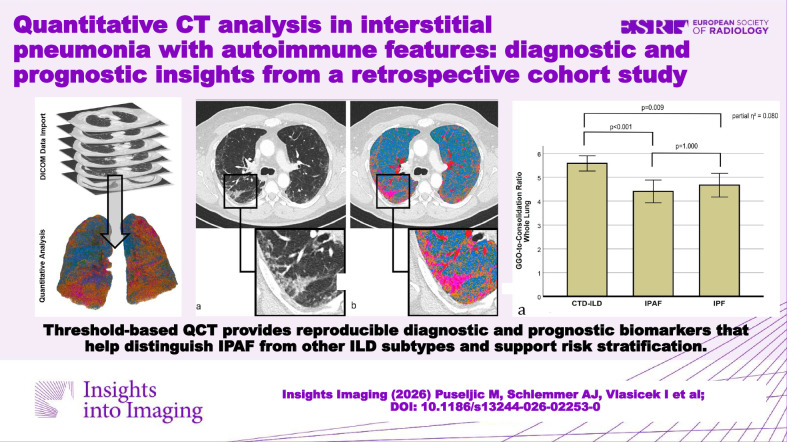

## Introduction

Interstitial pneumonia with autoimmune features (IPAF) is a subset of interstitial lung disease (ILD) characterized by clinical and serologic features of autoimmunity in the absence of a definitive rheumatologic diagnosis [1, 2]. The 2015 classification criteria introduced clinical, serologic, and morphologic domains to define IPAF, replacing some previous terms such as lung-dominant connective tissue disease [1]. There are conflicting findings regarding the placement of IPAF within the ILD spectrum, particularly between connective tissue disease-associated ILD (CTD-ILD) and idiopathic pulmonary fibrosis (IPF) [3]. While IPAF is generally associated with a better or similar prognosis compared to IPF [3–6], its prognosis relative to CTD-ILD has been reported as either worse or similar [7, 8] with overlapping clinical and demographic features [9].

Previous IPAF studies have relied on visual CT assessment, with most reporting nonspecific interstitial pneumonia (NSIP) as the predominant pattern [10–19], although NSIP/organizing pneumonia (OP), usual interstitial pneumonia (UIP) or probable UIP have also been described [20–23]. The presence of a UIP in IPAF has been identified as a predictor of disease progression with outcomes similar to IPF [14, 15], whereas other studies have demonstrated no significant prognostic impact [10, 21, 24]. Only one prior study has explored quantitative CT (QCT) in IPAF, reporting differences in Ground-glass opacities (GGO) and reticulation compared to CTD-ILD [25]. Evidence on the diagnostic and prognostic value of QCT in IPAF therefore remains limited.

The aim of this study was to evaluate the utility of threshold-based QCT for distinguishing IPAF from other ILD subtypes and to assess the prognostic relevance of specific QCT features. We hypothesize that patients with IPAF will show overlapping and distinct features in QCT analysis, progression-free survival (PFS), and QCT-derived risk factors.

## Materials and methods

### Study design

**Fig. 1:**
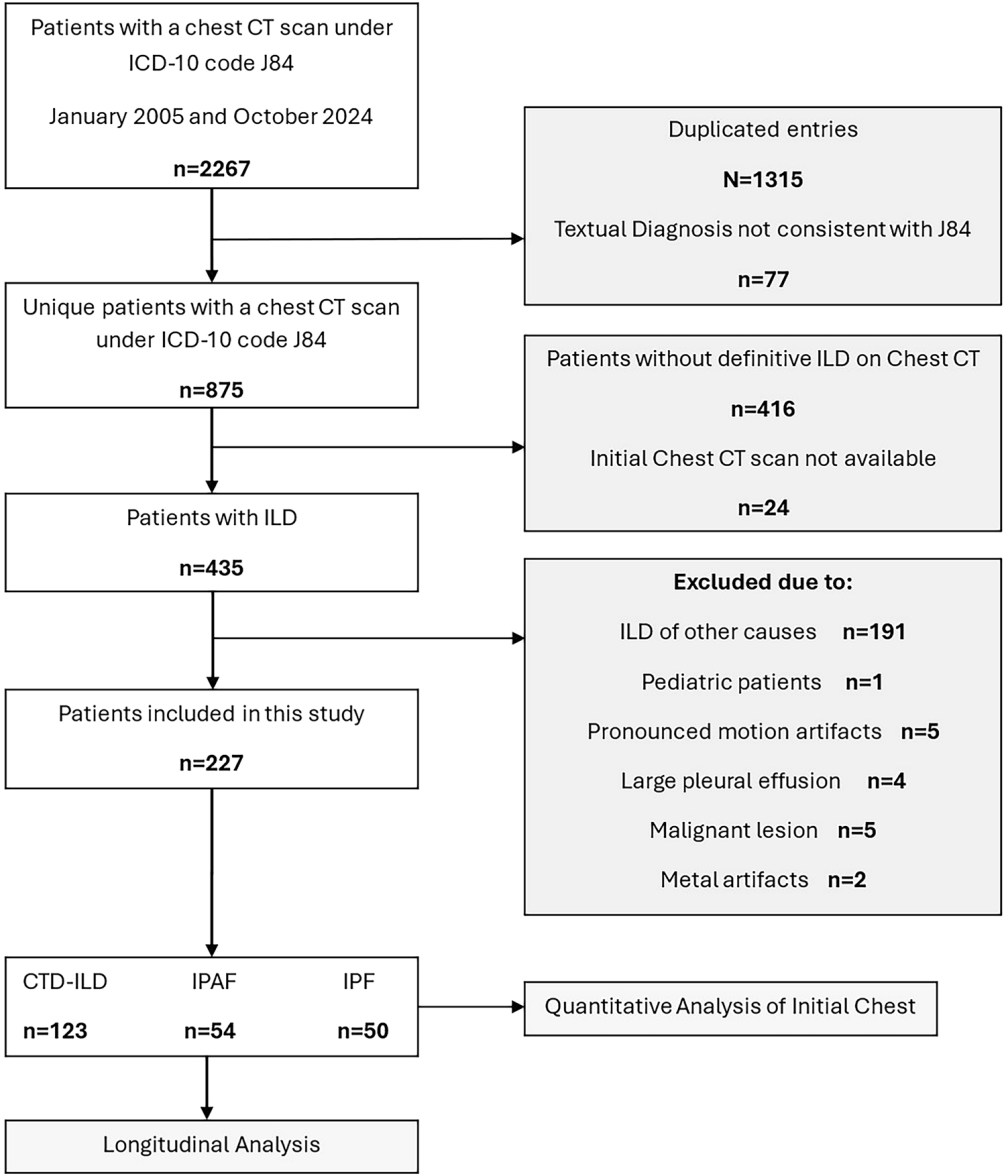
Patient flow diagram. ICD, International Classification of Diseases; CTD, connective tissue disease; ILD, interstitial lung disease; IPAF, interstitial pneumonia with autoimmune features; IPF, idiopathic pulmonary fibrosis; CT, computed tomography

This retrospective, single-center study received approval from the institutional ethics committee and adhered to the principles of the Declaration of Helsinki. The requirement for written informed consent was waived. Adult patients who underwent chest CT for suspected ILD between January 2005 and October 2024 were eligible. Inclusion criteria were: (1) the presence of parenchymal abnormalities consistent with ILD, (2) availability of the initial chest CT scan, and (3) availability of complete clinical data, serologic profile, and comprehensive multidisciplinary evaluation. The exclusion criteria were as follows: (1) ILD associated with any other cause than CTD, IPAF or IPF, (2) the presence of artifacts that could impact quantification, (3) neoplastic lesions, or (4) extensive pleural effusions (Fig. 1).

### Clinical and laboratory data

Clinical and laboratory data were obtained retrospectively from the hospital’s information system. Age at the time of CT scan, sex, smoking status, body mass index, hemoglobin levels, estimated glomerular filtration rate, lung function measurements, selected comorbidities, serologic profile [1, 12, 26], and treatment data were collected.

### Patient classification and longitudinal analysis

Each ILD case was reviewed in a multidisciplinary discussion and classified as having CTD-ILD, IPAF, or IPF. Patients classified as having ILD due to other causes were excluded. Patients between January 2005 and July 2015 who did not have a definitive diagnosis of CTD or IPF were retrospectively reviewed and reclassified as IPAF if they met the consensus definition [1]. The date of ILD diagnosis verified on a CT scan was set as the beginning of the time axis. Disease progression was verified clinically (relative decline of ≥ 10% in FVC% predicted and/or ≥ 15% in DLCO% predicted, acute exacerbation, or death attributable to ILD) [27], radiologically (interval increase in the extent of ILD/fibrosis), or by a combination of both. Patients who died unrelated to ILD were censored at the time of death.

### CT protocol and visual analysis

Chest CT scans were acquired using several CT scanners as volumetric acquisitions during full inspiration, in either the supine or prone position, with or without intravenous contrast media (CM). Detailed CT acquisition parameters are provided in Supplementary Tables S1, S2.

The ILD pattern was qualitatively assessed by two radiologists specialized in thoracic imaging and graded as UIP, probable UIP, indeterminate for UIP, NSIP, OP, NSIP/OP or lymphoid interstitial pneumonia (LIP) [12, 28]. Multicompartment involvement was assessed for the presence of pleural effusion or thickening, pericardial effusion or thickening or signs of airway disease [1]. Patient’s scan position, use of CM and the slice thickness of the lung kernel axial reconstruction were noted.

### Quantitative lung parenchyma analysis

**Fig. 2:**
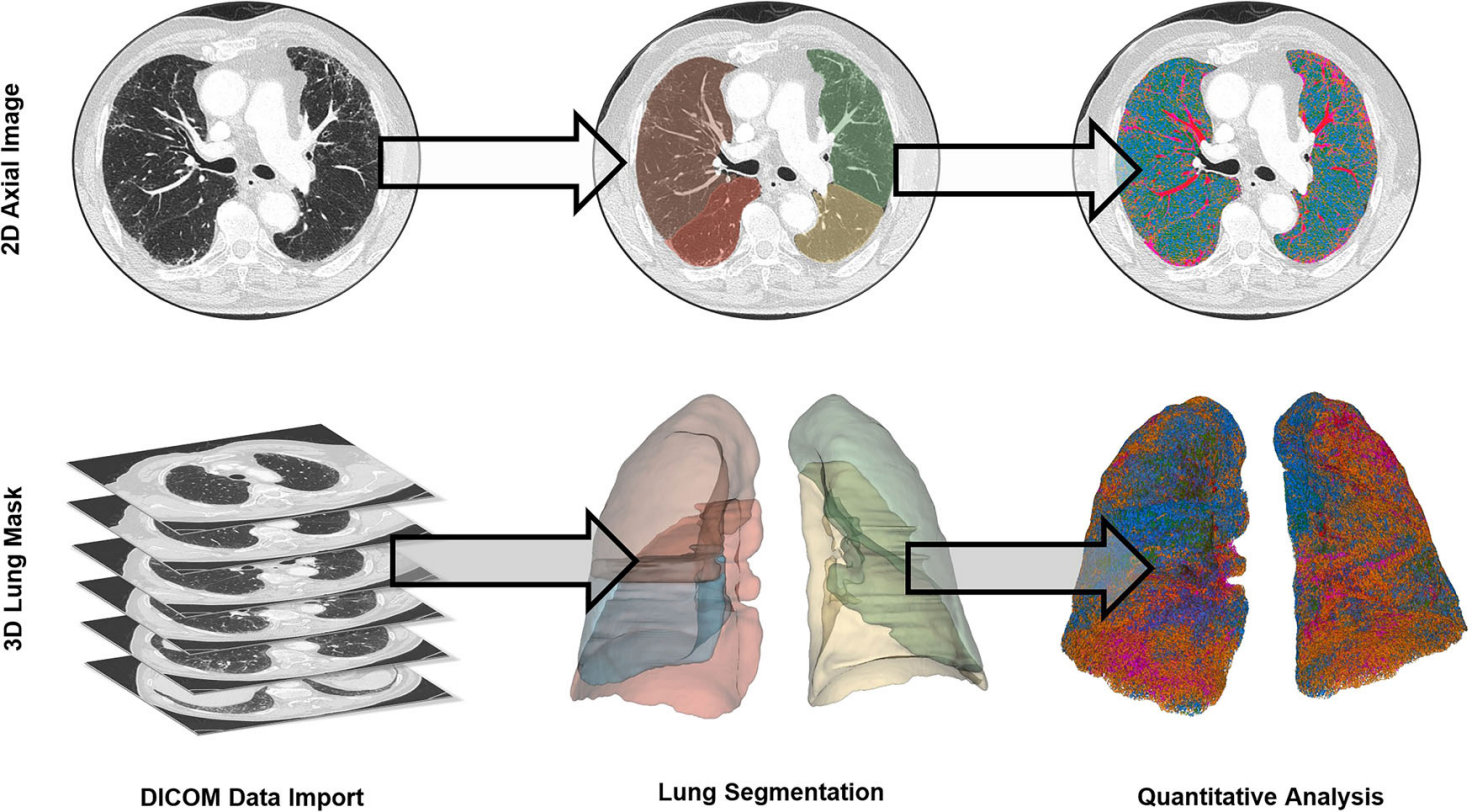
Schematic representation of the workflow from DICOM data import to quantitative lung parenchyma analysis. Axial CT images reconstructed using a lung kernel are imported into the Chest Imaging Platform (3D Slicer). A deep learning-based segmentation model is applied to segment each lobe and generate a 3D lung mask. Quantitative analysis is then performed using a threshold-based method, resulting in a color-coded 3D representation of lung parenchyma characteristics. The top row shows 2D axial slices at each step, while the bottom row presents the corresponding 3D reconstructions

**Fig. 3:**
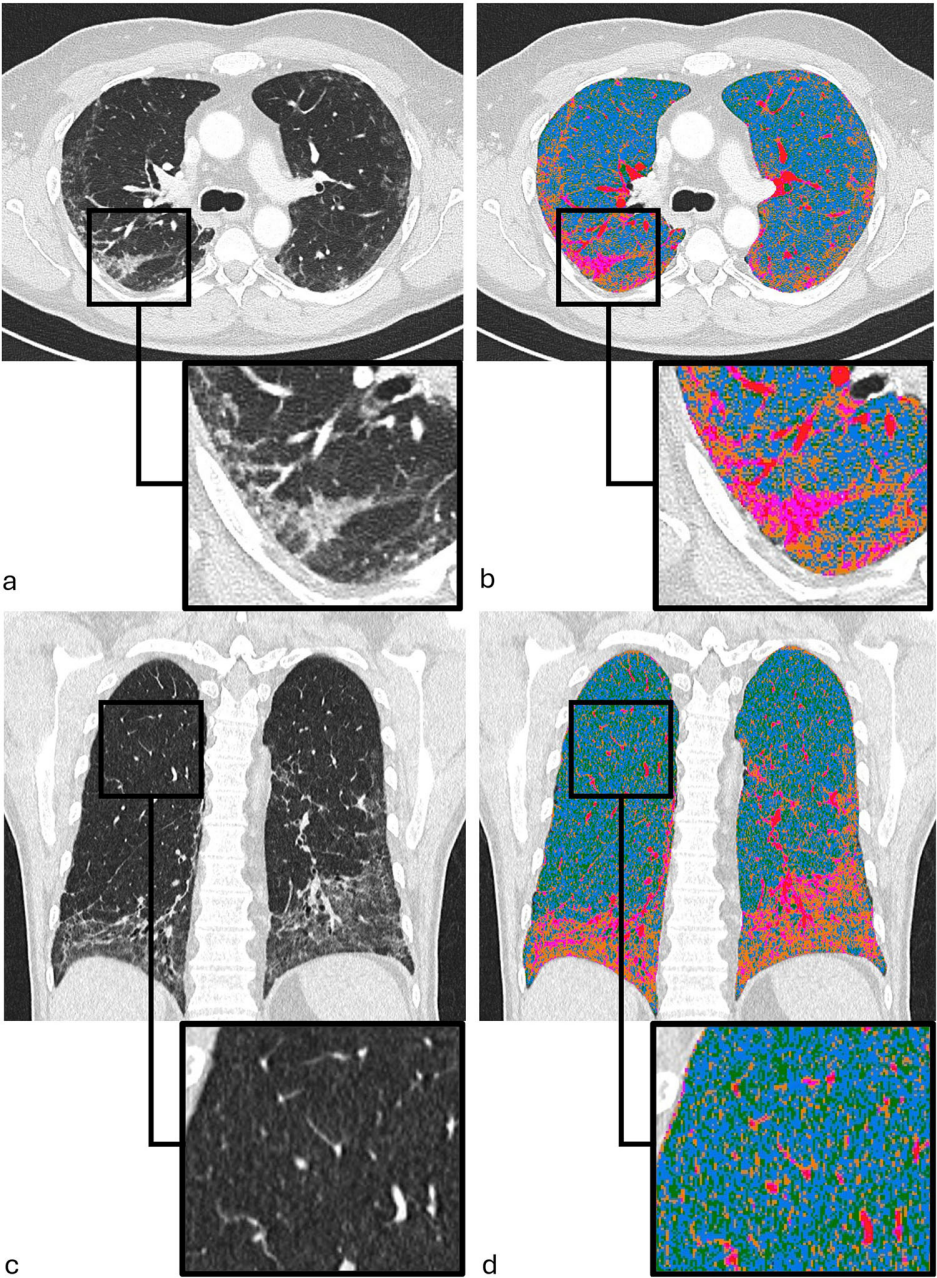
Example of threshold-based quantitative chest CT analysis outputs. **a** Axial chest CT image demonstrating visually evident consolidation and GGO with an enlarged view of the superior segment of the right lower lobe. **b** Corresponding output of the quantitative analysis, with an enlarged view of the same region. High-attenuation areas (consolidation) are color-coded in purple, GGO areas in orange, and normal lung parenchyma in blue. Intrapulmonary vessels are shown in red and are excluded from the final quantitative results. **c** Coronal CT reconstruction showing visually evident centrilobular emphysema, predominantly in the upper lung zones, with an enlarged view of the posterior segment of the right upper lobe. Consolidation and GGO are also seen in the lower lung regions. **d** Corresponding output of the quantitative analysis, with low-attenuation areas (emphysema) shown in green and normal lung in blue, along with an enlarged view of the posterior right upper lobe segment. CT, computed tomography; GGO, ground-glass opacity

DICOM data were processed using the Chest Imaging Platform (3D Slicer, version 5.6.2) [29]. The initial step involved creating a 3D lung mask from the axial lung kernel reconstruction images using LTRCLobes_R231, a U-Net-based, AI-driven deep learning model [30]. This was followed by volumetric, threshold-based quantitative analysis using the following HU thresholds: emphysema (−1050 to −950 HU), functional parenchyma (−950 to −750 HU), GGO (−750 to −400 HU), and consolidation (−400 to 0 HU). Affected lung parenchyma was calculated as the sum of GGO and consolidation. Intrapulmonary vessels were excluded from the analysis. Results were expressed as percentages and generated for the entire lung, right and left lungs, upper, middle, and lower thirds, ventral and dorsal regions, and individual lobes (Figs. 2, 3).

### Statistical analysis

Statistical analysis was performed using SPSS software (version 29.0; SPSS Inc.). A *p*-value < 0.05 was considered statistically significant. Data distribution was assessed using Q-Q plots and the Shapiro–Wilk test. Continuous variables are reported as mean ± standard deviation, while categorical variables are reported as frequencies and percentages. The Chi-squared test was used to compare categorical variables.

Robustness of the threshold-based QCT approach was assessed using a sensitivity analysis performed in a random subset of 50 patients (25 patients from 2005–2014 and 25 from 2015–2024) with ± 20 HU threshold variation for GGO and consolidation. Agreement with baseline values was evaluated using Spearman correlation and the intraclass correlation coefficient (ICC).

A General Linear Model was applied to compare continuous variables, adjusting for technical factors (slice thickness, CM application and scan position). Results are expressed as adjusted means, 95% confidence interval (CI), and *p*-values.

Kaplan–Meier survival analysis with log-rank test was employed to assess PFS. Univariable and multivariable Cox proportional hazards regression analyses were conducted to identify predictors of ILD progression. QCT variables were tested for multicollinearity using the variance inflation factor. To avoid multicollinearity (Supplementary Tables S3–S5), the following covariables were selected: total lung volume, percentage of emphysema and affected lung, and the GGO-to-consolidation ratio. Alternative models, including GGO and consolidation separately, were also evaluated. Statistically significant variables from the univariable analysis (*p* < 0.05) were included in the multivariable model, adjusted for age, sex, FEV₁% predicted, FVC% predicted, treatment and smoking status. Results are expressed as hazard ratios (HRs), 95% CIs, and *p*-values. Statistically significant predictors were additionally tested within subgroups in an exploratory approach, limited by the number of events and adjusted only for age and sex.

## Results

### Study group characteristics

A total of 227 patients were included, with a mean age of 63.6 ± 12.8 years, comprising 105 males (46.3%) and 122 females (53.7%). The sample consisted of 123 CTD-ILD (53.2%), 54 IPAF (23.8%), and 50 IPF (22.0%) patients. Patient characteristics are presented in Tables 1 and 2 and Supplementary Table 6.

**Table 1:** Patient demographics, clinical characteristics, treatment status and high-resolution CT ILD pattern features

Characteristic	Whole sample (*n* = 227)	CTD-ILD (*n* = 123)	IPAF (*n* = 54)	IPF (*n* = 50)	*p*-value
Sex (Male:Female)	105:122	29:94	35:19	41:9	**< 0.001**^**a,b**^
Age (years)	63.6 ± 12.7	60.8 ± 13.6	64.9 ± 11.8	68.9 ± 9.4	**< 0.001**^**b**^
BMI (kg/m^2^)	26.1 ± 4.4	25.1 ± 4.7	27.5 ± 4.2	27.1 ± 3.4	**0.001**^**a,b**^
Current or past smoker, no. (%)	98 (43.2)	39 (31.7)	31 (57.4)	28 (56.0)	**< 0.001**^**a,b**^
Arterial hypertension, no. (%)	83 (36.6)	40 (32.5)	19 (35.2)	24 (48.0)	0.162
Diabetes mellitus, no. (%)	25 (11.0)	5 (4.1)	6 (11.1)	14 (28.0)	**< 0.001**^**b,c**^
Coronary heart disease, no. (%)	46 (20.3)	13 (10.6)	16 (29.6)	17 (34.0)	**< 0.001**^**a,b**^
Dyslipidemia, no. (%)	29 (12.8)	12 (9.8)	9 (16.7)	8 (16.0)	0.329
COPD, no. (%)	14 (6.2)	4 (3.3)	7 (13.0)	3 (6.0)	0.050
Hemoglobin (g/dL)	13.9 ± 3.0	13.1 ± 1.8	14.7 ± 4.0	15.1 ± 3.6	**< 0.001**^**a,b**^
eGFR (mL/min)	77.0 ± 22.3	79.8 ± 21.2	77.6 ± 22.6	69.5 ± 23.5	**0.021**^**b**^
VC, % predicted	81.2 ± 22.1	86.0 ± 22.2	78.9 ± 22.7	71.9 ± 17.2	**< 0.001**^**a,b**^
FVC, % predicted	81.5 ± 22.1	86.1 ± 22.5	78.6 ± 22.5	73.1 ± 16.9	**0.001**^**b**^
FEV1, % predicted	80.7 ± 19.6	83.5 ± 20.2	78.3 ± 21.0	76.4 ± 15.6	0.055
FEV1/FVC	83.1 ± 12.5	82.8 ± 12.1	82.7 ± 13.9	82.2 ± 12.1	0.774
TLC, % predicted	78.5 ± 20.2	83.3 ± 19.8	75.5 ± 21.5	70.1 ± 16.5	**< 0.001**^**a,b**^
DL_CO_, % predicted	60.9 ± 20.7	67.1 ± 19.5	55.5 ± 19.6	51.5 ± 19.6	**< 0.001**^**a,b**^
Treatment status					**0.020**
None	108 (47.6)	45 (36.6)	29 (53.7)	34 (68.0)	**< 0.001**^**a,b**^
Anti-inflammatory	111 (48.9)	77 (62.6)	24 (44.4)	10 (20.0)	**< 0.001**^**a,b,c**^
Anti-fibrotic	8 (3.5)	1 (0.8)	1 (1.9)	6 (12.0)	**0.002**^**b,c**^
ILD pattern on initial CT					**< 0.001**
NSIP	111 (48.9)	78 (63.4)	27 (50.0)	6 (12.0)	**< 0.001**^**a,b,c**^
OP	4 (1.8)	3 (2.4)	1 (1.9)	0 (0.0)	0.812
NSIP/OP	11 (4.8)	4 (3.3)	7 (13.0)	0 (0.0)	**0.005**^**a,c**^
UIP	35 (15.4)	8 (6.5)	8 (14.8)	19 (38.0)	**< 0.001**^**b,c**^
Probable UIP	35 (15.4)	9 (7.3)	5 (9.3)	21 (42.0)	**< 0.001**^**b,c**^
Indeterminate for UIP	30 (13.2)	20 (16.3)	6 (11.1)	4 (8.0)	**0.316**
LIP	1 (0.4)	1 (0.8)	0 (0.0)	0 (0.0)	1.000

*CTD* connective tissue disease, *ILD* interstitial lung disease, *IPAF* interstitial pneumonia with autoimmune features, *IPF* idiopathic pulmonary fibrosis, *GGO* ground-glass opacity, *BMI* body mass index, *eGFR* estimated glomerular filtration rate, *VC* vital capacity, *FVC* forced vital capacity, *FEV₁* forced expiratory volume in 1 s, *TLC* total lung capacity, *DLCO* diffusion capacity of the lung for carbon monoxide, *NSIP* nonspecific interstitial pneumonia, *OP* organizing pneumonia, *UIP* usual interstitial pneumonia, *LIP* lymphoid interstitial pneumonia, *COPD* chronic obstructive pulmonary disease, *CT* computed tomography

^a^ Statistically significant difference between CTD-ILD and IPAF

^b^ Statistically significant difference between CTD-ILD and IPF

^c^ Statistically significant difference between IPAF and IPF

Bold values indicate statistical significance *p* < 0.05

**Table 2:** Characteristics of patients with interstitial pneumonia with autoimmune features, categorized by domain

Category	*n* = 54
Clinical domain	
Distal digital fissuring (i.e., “mechanic hands”)	1 (1.9)
Distal digital tip ulceration	2 (3.7)
Inflammatory arthritis or morning joint stiffness*	6 (11.2)
Palmar telangiectasia	0 (0.0)
Raynaud’s phenomenon	3 (5.6)
Unexplained digital edema	0 (0.0)
Gottron’s sign**	0 (0.0)
Serologic domain	
ANA ≥ 1:320 titer	31 (57.4)
Rheumatoid factor ≥ 2× upper limit of normal	9 (16.7)
Anti-CCP	5 (9.3)
Anti-dsDNA	4 (7.4)
Anti-Ro (SS-A)	10 (18.5)
Anti-La (SS-B)	0 (0.0)
Anti-ribonucleoprotein	2 (3.7)
Anti-Smith	1 (1.9)
Anti-topoisomerase (Scl.70)	2 (3.7)
Anti-tRNA synthetase	12 (22.2)
Anti-PM-Scl	0 (0.0)
Anti-MDA-5	1 (1.9)
ANCA***	6 (11.1)
Morphologic domain	
NSIP	27 (50.0)
OP	1 (1.9)
NSIP/OP overlap	7 (13.0)
LIP	0 (0.0)
UIP***	8 (14.8)
PROB UIP***	5 (9.3)
Indeterminate for UIP***	6 (11.1)
Multicompartment involvement, of which:	6 (11.1)
Unexplained pleural effusion or thickening	0 (0.0)
Unexplained pericardial effusion or thickening	0 (0.0)
Unexplained intrinsic airways disease	4 (7.4)
Unexplained pulmonary vasculopathy	2 (3.7)

*ANA* antinuclear antibodies, *CCP* cyclic citrullinated peptides, *MDA-5* melanoma differentiation-associated gene 5, *ANCA* antineutrophil cytoplasmic antibody, *NSIP* nonspecific interstitial pneumonia, *OP* organizing pneumonia, *LIP* lymphoid interstitial pneumonia, *UIP* usual interstitial pneumonia

* Defined as lasting ≥ 60 min

** Defined as an unexplained fixed rash on the digital extensor surfaces

*** Not included in the consensus definition of IPAF

IPF had a higher age at diagnosis (68.9 ± 9.4 years) compared with CTD-ILD (60.8 ± 13.6 years, *p* = 0.001), with no difference compared to IPAF (64.5 ± 11.8 years, *p* = 0.427) or between IPAF and CTD-ILD (*p* = 0.128). CTD-ILD group had a statistically significant female predominance (*n* = 94, 76.4%) in comparison to IPAF (*n* = 19, 35.2%, *p* < 0.001) and IPF (*n* = 9, 18%, *p* < 0.001), with no difference between IPAF and IPF (*p* = 0.076).

### ILD pattern

NSIP pattern was most commonly observed in CTD-ILD (*n* = 78, 63.4%) in comparison to IPAF (*n* = 27, 50.0%, *p* = 0.015) and IPF (*n* = 6, 12.0%, *p* < 0.001), with a statistically significant difference also between IPAF and IPF (*p* < 0.001).

UIP pattern was the most common in IPF (*n* = 19, 38.0%) in comparison to CTD-ILD (*n* = 8, 6.5%, *p* < 0.001) and IPAF (*n* = 8, 14.8%, *p* = 0.008), with no difference between IPAF and CTD-ILD (*p* = 0.091) (Table 1).

### Sensitivity analysis

Sensitivity analysis demonstrated high agreement between baseline and modified ± 20 HU thresholds for GGO, consolidation, and the GGO-to-consolidation ratio (r_s_ ≥ 0.99, *p* < 0.001; ICC ≥ 0.88, *p* < 0.001) (Supplementary Tables S7–S9).

### Quantitative lung parenchyma analysis

Percentage of consolidation for the whole lung was lower in CTD-ILD (6.2%, 95% CI: 5.0–7.4) compared to IPAF (8.9%, 95% CI: 7.1–10.7; *p* = 0.046), with no significant difference between CTD-ILD and IPF (8.2%, 95% CI: 6.3–10.1; *p* = 0.241) or between IPAF and IPF (*p* = 1.000).

**Fig. 4:**
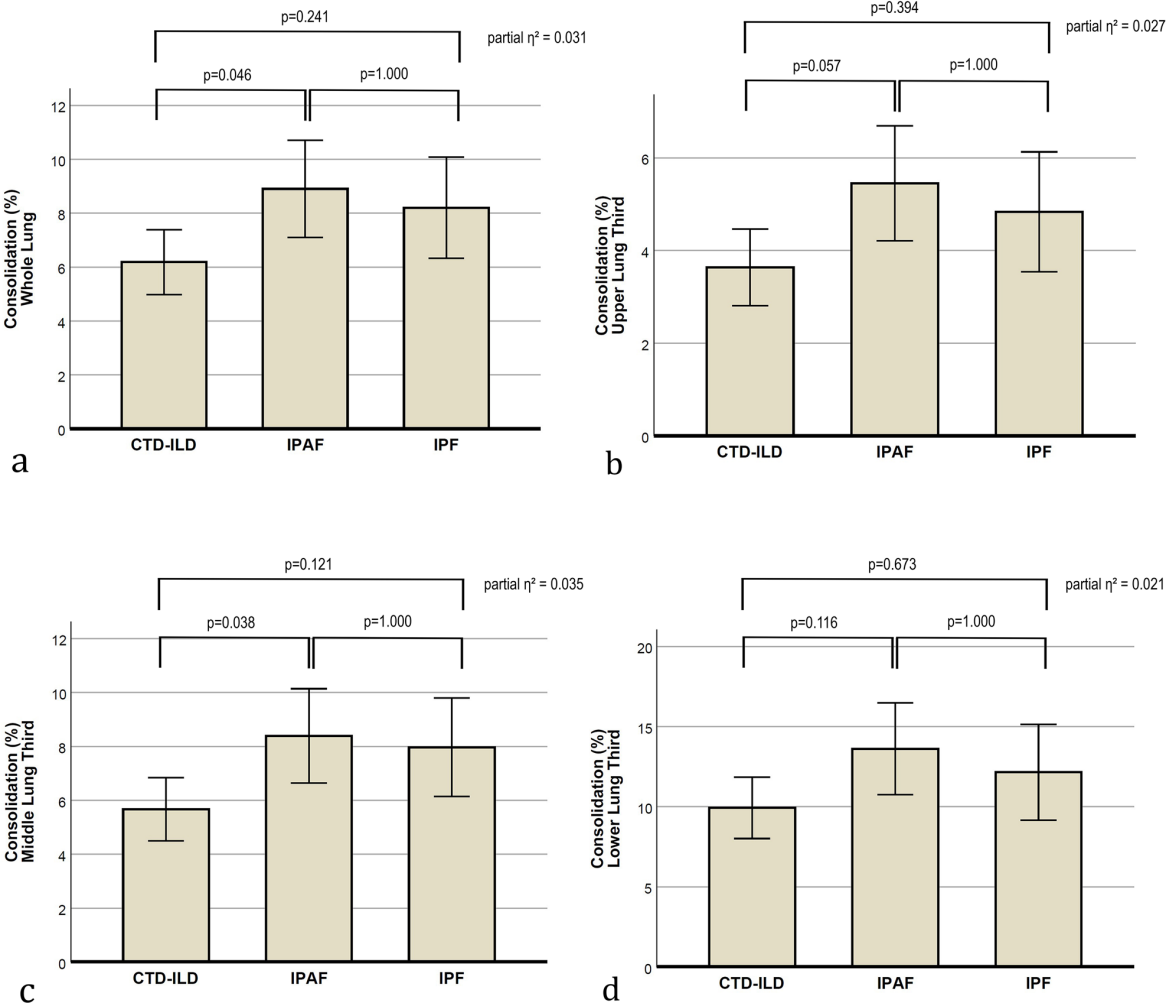
Bar plots presenting the adjusted means with 95% confidence intervals for the percentage of consolidation, based on the general linear model. *p*-values indicate between-group comparisons. The overall group effect size is also shown as partial η². Data are shown for (**a**) the whole lung, (**b**) the upper third, (**c**) the middle third, and (**d**) the lower third of the lung. CTD-ILD, connective tissue disease-associated interstitial lung disease; IPAF, interstitial pneumonia with autoimmune features; IPF, idiopathic pulmonary fibrosis

Consolidation percentages were also significantly different between CTD-ILD and IPAF in several lung compartments, for example middle lung third (5.7%, 95% CI: 4.5–6.8 vs. 8.4%, 95% CI: 6.6–10.1; *p* = 0.038) or right upper lobe (4.0%, 95% CI: 3.0–4.9 vs. 6.4%, 95% CI: 4.9–7.8; *p* = 0.029). Significant differences were also observed between CTD-ILD and IPF (e.g., ventral lung, 4.7%, 95% CI: 3.9–5.6 vs. 6.9%, 95% CI: 5.9–8.3; *p* = 0.026) (Fig. 4).

The GGO-to-consolidation ratio was higher in CTD-ILD (5.6, 95% CI: 5.3–5.9) than in IPAF (4.4, 95% CI: 3.9–4.9; *p* < 0.001) and IPF (4.7, 95% CI: 4.2–5.2; *p* = 0.009), with no difference between IPAF and IPF (*p* = 1.000).

**Fig. 5:**
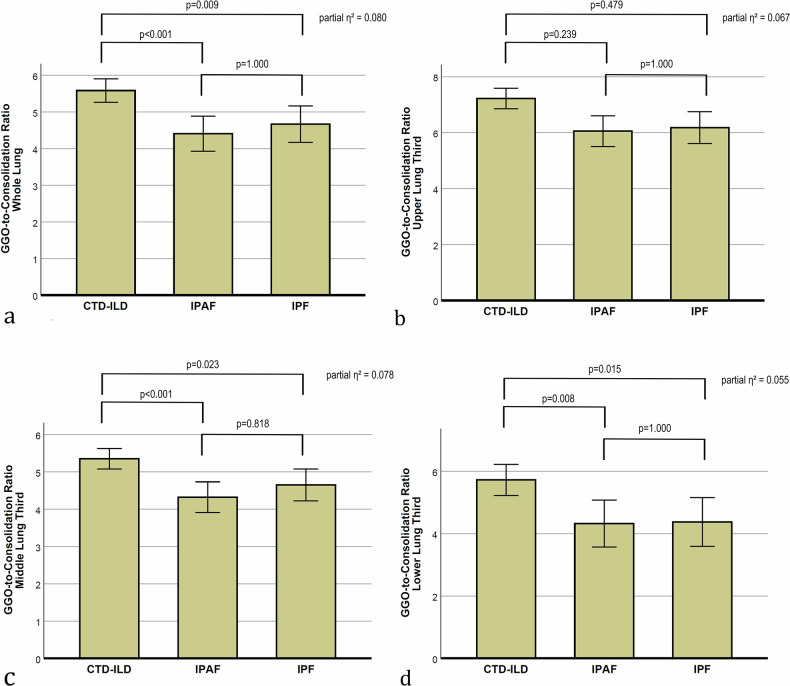
Bar plots presenting the adjusted means with 95% confidence intervals for the GGO-to-consolidation ratio, based on the general linear model. *p*-values indicate between-group comparisons. The overall group effect size is also shown as partial η². Data are shown for (**a**) the whole lung, (**b**) the upper third, (**c**) the middle third, and (**d**) the lower third of the lung. GGO, ground-glass opacity; CTD-ILD, connective tissue disease-associated interstitial lung disease; IPAF, interstitial pneumonia with autoimmune features; IPF, idiopathic pulmonary fibrosis

Similar results for GGO-to-consolidation ratio were seen in other lung compartments (Fig. 5), except for the middle (*p* = 0.280) and left lower lobe, where a significant difference was observed only between CTD-ILD (5.4, 95% CI: 5.0–5.8) and IPAF (4.2, 95% CI: 3.6–4.9; *p* = 0.012).

Complete results are presented in Table 3 and in Supplementary Table S10.

**Table 3:** Results of the general linear model between-group comparison, shown as adjusted means and 95% confidence intervals for the whole lung, upper, middle, and lower third, as well as for the ventral and dorsal lung parts

Parameter	CTD-ILD *n* = 123	IPAF *n* = 54	IPF *n* = 50	*p*-value
Whole lung				
Total volume, mL	4051.7 (3823.3, 4280.1)	3978.5 (3636.6, 4320.5)	4080.2 (3723.8, 4436.6)	0.910
Functional parenchyma, %	54.4 (51.8, 53.9)	50.1 (46.3, 54.0)	51.6 (47.6, 55.6)	0.177
Emphysema, %	11.6 (10.0, 13.2)	11.6 (9.2, 14.0)	9.1 (6.6, 11.6)	0.209
GGO, %	27.9 (25.5, 30.2)	29.4 (25.9, 32.9)	31.1 (27.5, 34.8)	0.339
Consolidation, %	6.2 (5.0, 7.4)	8.9 (7.1, 10.7)	8.2 (6.3, 10.1)	**0.032**^**a**^
Affected parenchyma, %	34.1 (30.7, 37.4)	38.4 (33.4, 43.4)	39.4 (34.2, 44.6)	0.169
GGO-to-consolidation ratio	5.6 (5.3, 5.9)	4.4 (3.9, 4.9)	4.7 (4.2, 5.2)	**< 0.001**^**a,b**^
Upper lung third				
Total volume, mL	995.4 (937.1, 1053.6)	982.5 (895.2, 1069.7)	1005.8 (914.9, 1096.7)	0.933
Functional parenchyma, %	74.4 (71.1, 77.7)	69.0 (64.1, 74.0)	70.0 (64.8, 75.1)	0.147
Emphysema, %	13.3 (11.5, 15.1)	13.9 (11.1, 16.6)	11.3 (8.5, 14.2)	0.390
GGO, %	22.5 (20.0, 25.1)	25.9 (22.0, 29.8)	25.0 (21.0, 29.0)	0.317
Consolidation, %	3.6 (2.8, 4.5)	5.5 (4.2, 6.7)	4.8 (3.5, 6.1)	**0.047**^**e**^
Affected parenchyma, %	26.2 (23.0, 29.4)	31.3 (26.4, 36.1)	29.9 (24.8, 35.0)	0.191
GGO-to-consolidation ratio	7.2 (6.9, 7.6)	6.1 (5.5, 6.6)	6.2 (5.6, 6.8)	**< 0.001**^**a,b**^
Middle lung third				
Total volume, mL	2047.0 (1935.5, 2158.5)	2007.3 (1840.4, 2174.2)	2050.7 (1876.8, 2224.6)	0.915
Functional parenchyma, %	69.3 (65.7, 72.8)	64.5 (59.2, 69.8)	63.1 (57.5, 68.6)	0.125
Emphysema, %	11.9 (10.6, 13.6)	11.9 (9.4, 14.4)	9.2 (6.6, 11.8)	0.191
GGO, %	28.4 (23.8, 28.9)	28.3 (24.6, 32.1)	30.1 (26.2, 34.1)	0.288
Consolidation, %	5.7 (4.5, 6.8)	8.4 (6.6, 10.1)	8.0 (6.1, 9.8)	**0.019**^**a**^
Affected parenchyma, %	32.0 (28.6, 35.5)	36.7 (31.5, 41.9)	38.1 (32.7, 43.5)	0.126
GGO-to-consolidation ratio	5.4 (5.1, 5.6)	4.3 (3.9, 4.7)	4.7 (4.2, 5.1)	**< 0.001**^**a,b**^
Lower lung third				
Total volume, mL	1009.3 (947.7, 1070.9)	988.8 (896.6, 1081.0)	1023.7 (927.6, 1119.8)	0.870
Functional parenchyma, %	54.8 (50.9, 58.6)	52.1 (46.3, 54.0)	51.6 (47.6, 55.6)	0.303
Emphysema, %	9.4 (8.0, 10.8)	8.6 (6.5, 10.7)	6.6 (4.4, 8.8)	0.113
GGO, %	36.1 (33.7, 38.6)	35.0 (31.5, 38.7)	39.1 (35.3, 42.8)	0.285
Consolidation, %	9.9 (8.0, 11.8)	13.6 (10.7, 16.5)	12.1 (9.1, 15.1)	0.100
Affected parenchyma, %	46.1 (42.4, 49.9)	48.6 (43.0, 54.2)	51.2 (45.4, 57.0)	0.355
GGO-to-consolidation ratio	5.7 (5.2, 6.2)	4.3 (3.6, 5.2)	4.4 (3.6, 5.1)	**0.002**^**a,b**^
Ventral lung				
Total volume, mL	1893.3 (1785.3, 2001.3)	1851.4 (1689.8, 2013.1)	1894.9 (1726.4, 2063.4)	0.904
Functional parenchyma, %	71.8 (68.6, 75.1)	66.8 (61.9, 71.7)	65.7 (60.6, 70.7)	0.080
Emphysema, %	12.8 (11.1, 14.6)	13.0 (10.4, 15.7)	10.1 (7.4, 12.9)	0.231
GGO, %	24.7 (22.2, 27.1)	27.4 (23.7, 31.0)	28.3 (24.4, 32.1)	0.233
Consolidation, %	4.7 (3.9, 5.6)	6.8 (5.5, 8.1)	6.9 (5.9, 8.3)	**0.007**^**a,b**^
Affected parenchyma, %	29.4 (26.2, 32.5)	34.2 (29.5, 39.0)	35.2 (30.3, 40.1)	0.087
GGO-to-consolidation ratio	5.7 (5.5, 6.1)	4.7 (4.3, 5.2)	4.9 (4.5, 5.3)	**< 0.001**^**a,b**^
Dorsal lung				
Total volume, mL	2158.4 (2037.5, 2279.3)	2127.1 (1946.1, 2308.1)	2127.1 (1946.1, 2308.1)	0.906
Functional parenchyma, %	62.7 (59.0, 66.4)	58.8 (53.3, 64.3)	57.4 (51.6, 63.1)	0.258
Emphysema, %	10.6 (9.1, 12.1)	10.3 (8.1, 12.6)	8.1 (5.7, 10.4)	0.200
GGO, %	30.7 (28.3, 33.2)	31.1 (27.5, 34.8)	33.7 (29.8, 37.5)	0.435
Consolidation, %	7.5 (5.9, 9.0)	10.7 (8.4, 13.2)	9.3 (6.9, 11.7)	0.067
Affected parenchyma, %	38.2 (34.5, 41.8)	41.9 (36.4, 47.2)	43.0 (37.3, 48.6)	0.303
GGO-to-consolidation ratio	5.6 (5.3, 6.0)	4.3 (3.7, 4.9)	4.6 (4.1, 5.2)	**< 0.001**^**a,b**^

*CTD* connective tissue disease, *ILD* interstitial lung disease, *IPAF* interstitial pneumonia with autoimmune features, *IPF* idiopathic pulmonary fibrosis, *GGO* ground-glass opacity

^a^ Statistically significant difference between CTD-ILD and IPAF

^b^ Statistically significant difference between CTD-ILD and IPF

^c^ Statistically significant difference between IPAF and IPF

^d^ No statistically significant difference in pairwise comparison

Bold values indicate statistical significance *p* < 0.05

### Progression-free survival

**Fig. 6:**
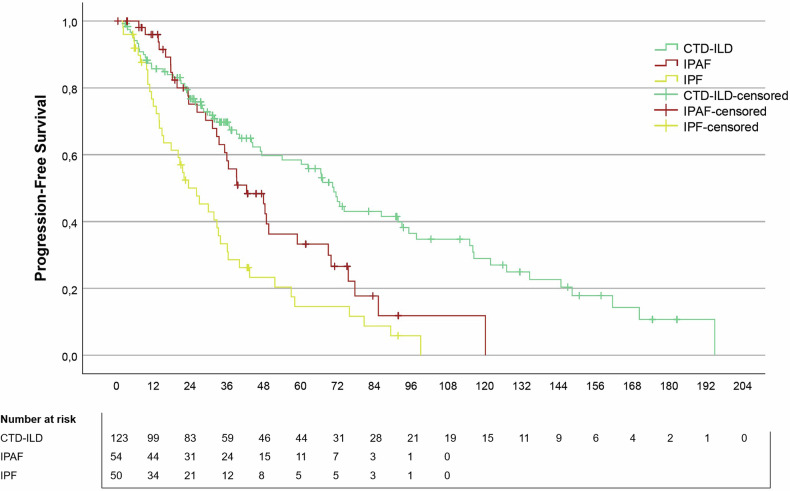
Kaplan–Meier curves illustrating progression-free survival in patients with CTD-ILD, IPAF, and IPF. Censored cases are indicated by tick marks. The number of patients at risk at each time point is shown below the plot. CTD-ILD, connective tissue disease-associated interstitial lung disease; IPAF, interstitial pneumonia with autoimmune features; IPF, idiopathic pulmonary fibrosis

The mean follow-up period was 53.3 ± 47.0 months for the CTD-ILD (73 events), 36.9 ± 26.9 months for IPAF (35 events), and 28.3 ± 24.5 months for IPF (45 events). The mean PFS was highest in the CTD-ILD group (80.3 months, 95% CI: 67.1–93.5 months) and was longer compared to the IPAF (49.8 months, 95% CI: 39.2–60.4 months, *p* = 0.015) and IPF (30.7 months, 95% CI: 23.2–38.2 months, *p* < 0.001). The mean PFS between IPAF and IPF was also statistically significantly different (*p* = 0.005) (Fig. 6).

### Prognostic factors

In the univariable analysis of whole-lung parameters, percentage of emphysema (HR = 0.98; 95% CI: 0.96–0.99; *p* = 0.008) and the GGO-to-consolidation ratio (HR = 0.80; 95% CI: 0.72–0.89; *p* < 0.001) were associated with a lower progression risk, while a higher percentage of affected lung with an increased risk (HR = 1.01; 95% CI: 1.00–1.02; *p* = 0.003). In the multivariable analysis, percentage of emphysema (HR = 0.96; 95% CI: 0.93–0.99; *p* = 0.011) and the GGO-to-consolidation ratio (HR = 0.85; 95% CI: 0.76–0.96; *p* = 0.010) remained significantly associated with a lower progression risk. However, the association with the percentage of affected lung was lost (HR = 0.99; 95% CI: 0.98–1.01; *p* = 0.190).

The UIP pattern was strongly associated with an increased risk in the univariable analysis (HR = 2.21; 95% CI: 1.43–3.40; *p* < 0.001). However, this association was no longer significant in the multivariable model for the whole lung (HR = 1.53; 95% CI: 0.97–2.42; *p* = 0.071), though it remained significant in multivariable models for several middle and lower lung regions (e.g., lower third, HR = 1.70; 95% CI: 1.07–2.71; *p* = 0.024).

Complete results are presented in Table 4, Supplementary Tables S11–S22. Results of alternative models are presented in Supplementary Tables S23–S28.

**Table 4:** Results of univariable and multivariable Cox regression analyses of quantitative CT data from the whole lung

	Univariable analysis		Multivariable analysis		
Variable	HR (95% CI)	*p*-value		HR (95% CI)	*p*-value
Sex	0.51 (0.37, 0.71)	< 0.001		0.63 (0.42, 0.93)	0.019
Age	1.03 (1.01, 1.04)	< 0.001		1.02 (1.01, 1.04)	0.002
FEV1% predicted	0.98 (0.97, 0.99)	< 0.001		0.99 (0.97, 0.99)	0.023
FVC% predicted	0.99 (0.98, 0.99)	< 0.001		1.00 (0.98, 1.01)	0.939
Current or past smoker	1.18 (0.85, 1.64)	0.315		0.85 (0.58, 1.24)	0.399
ILD treatment	0.81 (0.58, 1.1)	0.192		0.79 (0.56, 1.11)	0.181
Total lung volume	1.00 (1.00, 1.00)	0.440			
Percentage emphysema whole lung	0.98 (0.96, 0.99)	0.008		0.96 (0.93, 0.99)	0.007
Percentage affected whole lung	1.01 (1.00, 1.02)	0.003		0.99 (0.98, 1.01)	0.163
GGO-to-consolidation whole lung	0.84 (0.72, 0.89)	< 0.001		0.85 (0.75, 0.96)	0.007
Presence of UIP pattern	2.21 (1.43, 3.40)	< 0.001		1.53 (0.96, 2.41)	0.072

*HR* hazard ratio, *FEV₁* forced expiratory volume in 1 s, *FVC* forced vital capacity, *GGO* ground-glass opacity, *UIP* usual interstitial pneumonia

In the exploratory multivariable subgroup analysis, the GGO-to-consolidation ratio from the whole lung was associated with a lower progression risk in the CTD-ILD group (HR = 0.8; 95% CI: 0.7–1.0; *p* = 0.025), with no significant associations in IPAF or IPF (Table 5, Supplementary Table S29).

**Table 5:** Results of the exploratory univariable and multivariable Cox regression analysis of the GGO-to-consolidation ratio for the whole lung

Model	Variable	CTD-ILD *n* = 123		IPAF *n* = 54			IPF *n* = 50	
Univariable analysis	Variable	HR (95% CI)	*p*-value	HR (95% CI)	*p*-value	HR (95%CI)		*p*-value
	Age	1.02 (0.99, 1.03)	0.061	1.04 (1.00, 1.07)	0.024	1.02 (0.98, 1.06)		0.290
	Sex	0.51 (0.31, 0.86)	0.010	0.88 (0.43, 1.80)	0.732	1.41 (0.67, 2.96)		0.366
	GGO-to-consolidation	0.81 (0.70, 0.93)	**0.003**	0.86 (0.69, 1.06)	0.163	0.91 (0.72, 1.15)		0.425
Multivariable analysis	Age	1.01 (0.99, 1.03)	0.267	1.04 (1.00, 1.14)	0.047	1.02 (0.98, 1.06)		0.384
	Sex	0.55 (0.33, 0.92)	0.023	0.96 (0.47, 1.96)	0.906	1.41 (0.66, 2.99)		0.377
	GGO-to-consolidation	0.84 (0.73, 0.98)	**0.025**	0.91 (0.73, 1.14)	0.419	0.91 (0.72, 1.16)		0.446

The following numbers of events occurred during follow-up: CTD-ILD, 73; IPAF, 35; IPF, 45

*HR* hazard ratio, *CI* confidence interval, *CTD* connective tissue disease, *ILD* interstitial lung disease, *IPAF* interstitial pneumonia with autoimmune features, *IPF* idiopathic pulmonary fibrosis, *GGO* ground-glass opacity

Bold values indicate statistical significance *p* < 0.05

## Discussion

This study provides the first threshold-based QCT assessment of IPAF, with direct comparison to CTD-ILD and IPF. By quantifying both high- and low-attenuation lung components across multiple compartments, we identified the GGO-to-consolidation ratio as a novel imaging biomarker with diagnostic and prognostic relevance. Quantitatively, IPAF exhibited a parenchymal profile more closely aligned with IPF than with CTD-ILD, showing no significant QCT differences from IPF but consistently greater consolidation than CTD-ILD. The GGO-to-consolidation ratio was consistently higher in CTD-ILD than in IPAF and IPF. A higher GGO-to-consolidation ratio and greater emphysema extent were independently associated with reduced risk of progression, while the presence of a UIP pattern predicted worse outcomes. In the exploratory subgroup analysis, the GGO-to-consolidation ratio was associated with reduced progression risk only in CTD-ILD. Some demographic differences were also identified.

Threshold-based QCT is a well-established method for assessing ILD severity based on pixel counts within predefined attenuation thresholds corresponding to structural parenchymal abnormalities [10]. Although there are no definitive cut-off values, the thresholds applied in our study align with several prior studies [31, 32]. Given that IPAF shares established ILD imaging phenotypes, such as UIP or NSIP, which do not differ from those observed in other ILD types [1, 22], the application of threshold-based QCT in IPAF is reasonable.

Since the introduction of IPAF classification criteria in 2015, several studies have attempted to categorize this patient group and refine the current classification [1, 2]. Demographic findings remain inconsistent, with some studies reporting a younger or similar [5, 8, 11] age at ILD onset in IPAF compared to IPF, or a similar age to CTD-ILD [8, 9]. Most studies noted a female predominance in IPAF compared to IPF [4, 5, 16, 25] and either similar [5, 9] or lower rates than in CTD-ILD [25]. Reports on ILD patterns have also varied. NSIP is frequently described in IPAF [4, 7, 10–19], although other studies reported NSIP/OP [20], probable UIP, or UIP as more common [21–23]. Although UIP is not included in the morphologic domain [1], the term UIP-associated IPAF has been described in recent studies [13–15, 21, 22]. Our results did not identify age or sex differences between IPAF and IPF, whereas the CTD-ILD group showed a greater female predominance compared to both IPAF and IPF. The ILD pattern differed significantly across groups, with NSIP being most common in CTD-ILD and UIP in IPF.

In previous ILD studies, consolidation or high-attenuation areas have been associated with reduced lung function and higher mortality [33], whereas the presence of GGO has been linked to active inflammation, better prognosis and treatment response [34]. Patients with IPAF were found to have more extensive GGO and consolidation than IPF, despite no difference in the overall distribution of lung changes [20]. Only one study has performed QCT analysis in patients with IPAF, showing higher volumes of GGO and reticulation compared to CTD-ILD, although consolidation or high-attenuation areas were not analyzed [25]. Our study identified a difference in consolidation percentage between CTD-ILD and IPAF. In addition, the GGO-to-consolidation ratio highlighted a more pronounced distinction between CTD-ILD and both IPAF and IPF, while no difference was observed between IPAF and IPF. These results may reflect a shared fibrotic disease phenotype of IPAF and IPF. However, certain subsets of IPAF, such as those with non-UIP patterns or different domain characteristics, may exhibit distinct behavior, which limits the generalizability of the IPAF group toward IPF [19–22].

IPAF is generally associated with better outcomes compared to IPF, but worse than CTD-ILD [3, 11]. Prognosis appears to be influenced by the presence of UIP, which has been linked to poorer outcomes similar to IPF [3, 6, 15, 21]. However, other studies found no significant survival difference between IPAF and IPF [3, 4, 13], even in the presence of UIP [14]. One QCT study found that increased reticulation and GGO were associated with higher mortality in IPAF [25]. Although not specific to IPAF, some studies have linked the presence of emphysema to better survival [31], potentially reflecting distinct ILD phenotypes, interactions between emphysematous and fibrotic processes, or altered functional profiles [31, 32]. In our study, patients with IPAF demonstrated intermediate PFS, better than IPF but worse than CTD-ILD. A higher GGO-to-consolidation ratio and emphysema were associated with better prognosis, especially for the middle and lower lung compartments. The presence of a UIP pattern was associated with a worse prognosis, particularly in the mid and lower lung zone models. Interestingly, a higher GGO-to-consolidation ratio was linked to better prognosis only in the CTD-ILD subgroup. However, this observation should be interpreted with caution due to the limited number of subgroup events and the exploratory nature of the analysis.

The GGO-to-consolidation ratio may serve as a complementary and independent metric in the evaluation of ILD patients. Our results demonstrated potential in discriminating different ILD phenotypes, as well as serving as a composite imaging biomarker incorporating GGO and consolidation and potentially identifying high-risk patients requiring closer monitoring. Furthermore, differential behavior was observed in multivariable models compared with the UIP pattern, suggesting additional prognostic value beyond visual ILD pattern assessment.

This study has several limitations. First, it was retrospective and conducted at a single tertiary care center, which may limit generalizability. Second, threshold-based QCT measurements may be influenced by scanner differences, acquisition protocols, reconstruction kernels, and patient-related factors such as inspiratory depth [35]. Although our analyses were adjusted for technical parameters, and all analyses were performed using high-frequency (lung) reconstruction kernels, residual variability related to acquisition heterogeneity cannot be excluded. However, sensitivity analysis demonstrated high robustness of the applied QCT approach. Third, the subgroup sample size and the number of progression events were modest and imbalanced, reducing the statistical power. Finally, the threshold-based QCT approach used in this study does not allow quantitative differentiation of reticulation and honeycombing, which may limit assessment of fibrotic subcomponents.

In conclusion, threshold-based QCT provides a reproducible and relevant framework for evaluating IPAF. QCT features and progression patterns positioned IPAF closer to IPF than CTD-ILD. The GGO-to-consolidation ratio emerged as a promising biomarker for disease differentiation and prognosis.

## Supplementary information

ELECTRONIC SUPPLEMENTARY MATERIAL

## Data Availability

The dataset generated and analyzed during the study is available from the corresponding author upon reasonable request.

## Abbreviations

CI: Confidence interval
CM: Contrast media
CTD: Connective tissue disease
CTD-ILD: Connective tissue disease-associated interstitial lung disease
DLCO: Diffusion capacity of the lung for carbon monoxide
FEV₁: Forced expiratory volume in 1 s
FVC: Forced vital capacity
GGO: Ground-glass opacity
HR: Hazard ratio
ILD: Interstitial lung disease
IPAF: Interstitial pneumonia with autoimmune features
IPF: Idiopathic pulmonary fibrosis
LIP: Lymphoid interstitial pneumonia
NSIP: Nonspecific interstitial pneumonia
OP: Organizing pneumonia
PFS: Progression-free survival
QCT: Quantitative computed tomography
UIP: Usual interstitial pneumonia
