# Supplementary material for: Quantitative CT analysis in interstitial pneumonia with autoimmune features: diagnostic and prognostic insights from a retrospective cohort study
Source: Insights Imaging. 2026 Mar 18;17:76. doi: 10.1186/s13244-026-02253-0 (PMC13000105; doi:10.1186/s13244-026-02253-0)
Supplement: Supplementary file 1 — ELECTRONIC SUPPLEMENTARY MATERIAL [file 13244_2026_2253_MOESM1_ESM.pdf]

Quantitative CT Analysis in Interstitial Pneumonia with Autoimmune Features: Diagnostic and Prognostic Insights from a Retrospective Cohort Study

Electronic Supplementary Material

Insights Imaging (2026) Puseljic M, Schlemmer AJ, Vlasicek I, et al.

Scan parameter	*GE Revolution*	*Siemens Sensation Cardiac 64*	*SOMATOM Force*	*SOMATOM Definition Flash*	*SOMATOM Perspective*	*SOMATOM Definition AS+*
Scan type	Helical	Helical	Helical	Helical	Helical	Helical
Direction	Craniocaudal	Craniocaudal	Craniocaudal	Craniocaudal	Craniocaudal	Craniocaudal
Inspiration/Expiration	Inspiration	Inspiration	Inspiration	Inspiration	Inspiration	Inspiration
Detector row	256	32	96	96	64	64
Slices per rotation	256	64	192	192	128	64
Kernel reconstruction	HD Lung DETAIL2	B70f B41f	Bl64d\3 Br40d\3	I70f\3 I31f\3	B70s I41s\3	Br51f\2 Bf37f\3
Tube current	Modulated	Modulated	Modulated	Modulated	Modulated	Modulated
Tube voltage	100-255 kv	120 kv	90 – 110 kv	100 kv	110 – 130 kv	100 - 120 kv
Pitch	0.992	1.399	0.599	1.200	1.000	1.000
Gantry tilt	0	0	0	0	0	0
Collimation	80 mm	60 mm	60 mm	60 mm	60 mm	60 mm
Matrix	512 x 512	512 x 512	512 x 512	512 x 512	512 x 512	512 x 512

Supplementary Table S1. Scan parameters from each included CT scanner.

Scan parameter	*Toshiba Aquilion 64*	*Somatom Definition*	*Somatom Definition AS*	*Siemens Sensation 64*	*Philips Ingenuity Core 128*
Scan type	Helical	Helical	Helical	Helical	Helical
Direction	Craniocaudal	Craniocaudal	Craniocaudal	Craniocaudal	Craniocaudal
Inspiration/Expiration	Inspiration	Inspiration	Inspiration	Inspiration	Inspiration
Detector row	64	32	64	32	64
Slices per rotation	64	64	128	64	128
Kernel reconstruction	FC86	B80f	I70f\3	B70f	YA
	FC15	B31f	I31f\3	B41f	B
Tube current	Modulated	Modulated	Modulated	Modulated	Modulated
Tube voltage	120 kv	120 kv	100 kv	120 kv	120 kv
Pitch	0.828	1.200	1.200	1.200	1.015
Gantry tilt	0	0	0	0	0
Collimation	40 mm	50 mm	50 mm	50 mm	50 mm
Matrix	512 x 512	512 x 512	512 x 512	512 x 512	512 x 512

Supplementary Table S1. Continued.

Parameter	*No. (%)*
Whole study sample	**n=227**
Patient scan position	
Supine	209 (92.1)
Prone	18 (7.9)
Contrast administration	
Nonenhanced	107 (47.1)
Contrast-enhanced	120 (52.9)
Contrast enhanced scans	***n=120***
Contrast phase	
Pulmonary artery angiography (Bolus tracking)	30 (25.0)
Fixed Delay, 25s	89 (74.2)
Fixed Delay, 70s	1 (0.8)
Patient scan position	
Supine	118 (98.3)
Prone	2 (1.7)

Supplementary Table S2. Summary of scan parameters according to scan position and contrast phase. Contrast-enhanced scans were performed after intravenous CM administration using a fixed delay in the arterial phase (25 s delay) or venous phase (70 s delay), or as CT pulmonary angiography with bolus tracking set in the truncus pulmonalis. CM = contrast media

Variable	Unstandardized Coefficients		Standardized Coefficients		P-value		
	B	Std. Error	Beta			Tolerance	VIF
Total lung volume (ml)	0.001	0.001	0.057	0.674	0.501	0.540	1.85
Volume functional lung (%)	3.860	4.120	2.854	0.937	0.350	0.000	2444.94
Volume emphysema (%)	3.947	4.122	1.986	0.957	0.339	0.001	1133.22
Volume GGO (%)	5.395	5.739	3.661	0.940	0.348	0.000	3995.83
Volume consolidation (%)	6.059	5.730	2.096	1.058	0.291	0.001	1034.71
Volume affected lung (%)	-2.048	4.391	-1.969	-0.466	0.641	0.000	4693.48
GGO-to-consolidation ratio	0.913	1.002	.086	0.911	0.363	0.425	2.35

Supplementary Table S3. Results of a multiple linear regression used to assess multicollinearity, including all parameters from the quantitative chest CT analysis. This model was therefore dismissed. The results indicate high multicollinearity when all quantitative parameters are included in the multivariable model. GGO = ground-glass opacity; VIF = variance inflation factor

Variable	Unstandardized Coefficients		Standardized Coefficients		P-value		
	B	Std. Error	Beta			Tolerance	VIF
GGO (%)	266.612	281.209	2.916	0.948	0.344	0.000	2256.492
Consolidation (%)	180.225	282.853	1.005	0.637	0.525	0.002	593.083
Affected lung parenchyma (%)	-244.872	281.575	-3.794	-0.870	0.385	0.000	4540.262

Supplementary Table S4. Results of a multiple linear regression assessing multicollinearity, including a combination of variables with high VIF values indicating strong multicollinearity. This model was therefore dismissed. GGO – ground-glass opacity; VIF – variance inflation factor

Variable	Unstandardized Coefficients		Standardized Coefficients		P-value		
	B	Std. Error	Beta			Tolerance	VIF
Total lung volume (ml)	0.001	0.001	0.067	0.809	0.420	0.556	1.800
Volume emphysema (%)	0.132	0.176	0.066	0.750	0.454	0.485	2.063
Volume affected lung (%)	-0.309	0.111	-0.297	-2.793	0.006	0.336	2.977
GGO-to-consolidation ratio	0.154	0.782	0.015	0.197	0.844	0.697	1.435

Supplementary Table S5. Results of multiple linear regression assessing multicollinearity, including only variables from quantitative chest CT with acceptable VIF values. *GGO* – ground-glass opacity; *VIF* – variance inflation factor

CTD-ILD Group (n=123)	*Age*	*Male:Female*	*UIP Pattern*	*NSIP Pattern*
Rheumatoid arthritis (n=20)	65.9 ± 9.5	8:12	3 (15.0)	8 (40.0)
Systemic lupus erythematosus (n=9)	47.0 ± 18.7	1:8	0 (0.0)	6 (66.7)
Systemic sclerosis (n=74)	61.7 ± 13.0	16:58	4 (5.4)	56 (75.7)
Sjogren's syndrome (n=3)	73.8 ± 15.7	0:3	0 (0.0)	1 (33.3)
Polymyositis/Dermatomyositis (n=1)	43	1:0	0 (0.0)	0 (0.0)
Mixed connective tissue disease (n=5)	53.0 ± 12.4	0:5	0 (0.0)	3 (60.0)
Antisynthetase syndrome (n=11)	58.7 ± 10.9	3:8	1 (9.1)	4 (36.4)

Supplementary Table S6. Composition of the CTD-ILD group. CTD – connective tissue disease; ILD – interstitial lung disease; UIP – usual interstitial pneumonia; NSIP – non-specific interstitial pneumonia.

Parameter	*Mean ± SD*	*Median [IQR]*
GGO −20 HU	3.25 ± 1.44 %	3.20 [2.00]
GGO +20 HU	3.11 ± 1.44 %	2.90 [2.12]
Consolidation −20 HU	1.00 ± 0.94 %	0.65 [1.13]
Consolidation +20 HU	0.51 ± 0.64 %	0.20 [0.53]
GGO-to-consolidation ratio −20 HU	0.48 ± 0.43	0.38 [0.53]
GGO-to-consolidation ratio +20 HU	0.89 ± 0.62	0.73 [0.86]

Supplementary Table S7. Results of the sensitivity analysis of quantitative CT metrics under ±20 HU threshold variation, presented as absolute deviations from baseline threshold values. Data are shown as mean ± standard deviation and median with interquartile range. GGO = ground-glass opacity; SD = standard deviation; IQR = interquartile range

Parameter	Correlation Coefficient (r_s)	p-value
GGO		
Baseline vs +20 HU	0.998	< 0.001
Baseline vs −20 HU	0.995	< 0.001
+20 HU vs −20 HU	0.993	< 0.001
Consolidation		
Baseline vs +20 HU	0.992	< 0.001
Baseline vs −20 HU	0.992	< 0.001
+20 HU vs −20 HU	0.999	< 0.001
GGO-to-consolidation ratio		
Baseline vs +20 HU	0.989	< 0.001
Baseline vs −20 HU	0.989	< 0.001
+20 HU vs −20 HU	0.996	< 0.001

Supplementary Table S8. Results of the sensitivity analysis presented as spearman correlation analysis of quantitative CT metrics across baseline and modified ±20 HU threshold variation. Correlation coefficients are shown for whole-lung GGO, consolidation, and the GGO-to-consolidation ratio comparing baseline thresholds with −20 HU and +20 HU variations (n = 50). GGO = ground-glass opacity

Model	ICC	95% CI	F	df1 / df2	p-value

GGO					
Single measures	0.938	0.474 – 0.981	290.0	49 / 98	< 0.001
Average measures	0.979	0.730 – 0.994	290.0	49 / 98	< 0.001
Consolidation					
Single measures	0.983	0.945 – 0.993	305.3	49 / 98	< 0.001
Average measures	0.994	0.981 – 0.998	305.3	49 / 98	< 0.001
GGO-to-consolidation ratio					
Single measures	0.876	0.600 – 0.948	46.6	49 / 98	< 0.001
Average measures	0.955	0.818 – 0.982	46.6	49 / 98	< 0.001

Supplementary Table S9. Results of the sensitivity analysis presented as intraclass correlation analysis of quantitative CT metrics across baseline and modified attenuation thresholds (±20 HU). Intraclass correlation coefficients were calculated using a two-way mixed-effects model with absolute agreement for whole-lung GGO, consolidation, and the GGO-to-consolidation ratio (n = 50). GGO = Ground-glass opacity; CI = confidence interval

Parameter	CTD-ILD	IPAF	IPF	P-value
**Right upper lobe**				
Total volume, ml	971.9 (914.5, 1029.4)	1028.1 (942.0, 1114.1)	1007.2 (917.5, 1096.9)	0.546
Functional parenchyma, %	73.2 (69.8, 76.5)	66.6 (61.6, 71.6)	66.8 (61.6, 72.1)	0.044 [d]
Emphysema, %	13.1 (11.3, 14.9)	12.9 (10.3, 15.6)	10.5 (7.7, 13.3)	0.273
GGO, %	22.9 (20.3, 25.5)	27.1 (23.1, 31.0)	27.1 (23.0, 31.1)	0.118
Consolidation, %	4.0 (3.0, 4.9)	6.4 (4.9, 7.8)	6.1 (4.5, 7.6)	**0.012** [a]
Affected parenchyma, %	26.8 (23.5, 30.2)	33.4 (28.4, 38.4)	33.2 (28.0, 38.4)	**0.044** [d]
GGO-to-consolidation ratio	14.2 (13.5, 14.9)	11.5 (10.4, 12.6)	11.8 (10.6, 12.9)	**< 0.001** [a, b]
**Middle lobe**				
Total volume, ml	334.8 (298.4, 371.3)	393.1 (338.6, 447.7)	352.4 (295.5, 409.2)	0.225
Functional parenchyma, %	69.4 (66.1, 72.8)	63.3 (58.3, 68.3)	64.8 (59.5, 70.0)	0.100
Emphysema, %	13.0 (11.2, 14.7)	12.0 (9.4, 14.6)	9.9 (7.2, 12.7)	0.195
GGO, %	25.7 (23.2, 28.3)	27.8 (23.9, 31.6)	28.0 (24.0, 32.1)	0.546
Consolidation, %	5.0 (4.1, 5.9)	6.9 (5.5, 8.2)	7.1 (5.7, 8.5)	**0.017** [b]
Affected parenchyma, %	30.7 (27.4, 34.0)	34.6 (29.7, 39.6)	35.1 (30.0, 40.3)	0.256
GGO-to-consolidation ratio	6.0 (2.8, 9.1)	4.6 (0.2, 9.4)	9.9 (4.9, 14.9)	0.280
**Right lower lobe**				
Total volume, ml	895.1 (828.1, 962.1)	841.9 (741.6, 942.2)	835.6 (731.1, 940.2)	0.547
Functional parenchyma, %	56.0 (52.1, 59.9)	52.4 (46.6, 58.2)	50.4 (44.4, 56.5)	0.281
Emphysema, %	9.4 (8.0, 10.8)	8.8 (6.6, 11.0)	6.6 (4.4, 8.9)	0.132
GGO, %	33.3 (31.8, 36.9)	33.8 (29.9, 37.6)	37.1 (33.1, 41.1)	0.420
Consolidation, %	9.6 (7.7, 11.6)	12.0 (9.0, 14.9)	12.5 (9.4, 15.5)	0.238
Affected parenchyma, %	44.0 (40.1, 47.9)	45.7 (39.9, 51.5)	49.6 (43.6, 55.7)	0.316
GGO-to-consolidation ratio	10.2 (9.4, 11.0)	7.7 (6.5, 8.8)	8.1 (6.9, 9.3)	**< 0.001** [a, b]
**Left upper lobe**				
Total volume, ml	1055.7 (990.5, 1120.9)	1073.1 (975.5, 1170.8)	1067.8 (966.1, 1169.6)	0.954
Functional parenchyma, %	69.8 (66.5, 73.2)	65.2 (60.2, 70.2)	64.4 (59.2, 69.6)	0.145
Emphysema, %	12.8 (11.1, 14.6)	13.0 (10.4, 15.7)	10.2 (7.4, 12.9)	0.221
GGO, %	24.5 (21.9, 27.0)	26.7 (22.9, 30.6)	28.6 (24.6, 32.6)	0.224
Consolidation, %	4.8 (3.7, 6.0)	7.7 (6.0, 9.5)	6.5 (4.7, 8.3)	**0.021** [a]
Affected parenchyma, %	29.3 (25.9, 32.8)	34.5 (29.3, 39.6)	35.1 (29.7, 40.5)	0.120
GGO-to-consolidation ratio	5.9 (5.6, 6.2)	4.8 (4.3, 5.2)	5.1 (4.7, 5.6)	**< 0.001** [a, b]
**Left lower lobe**				
Total volume, ml	806.3 (739.9, 872.7)	828.1 (728.7, 927.4)	808.1 (704.6, 911.7)	0.935
Functional parenchyma, %	56.8 (52.7, 60.9)	54.4 (48.3, 60.5)	51.7 (45.4, 58.1)	0.423
Emphysema, %	9.5 (8.0, 11.0)	9.3 (7.1, 11.5)	7.0 (4.7, 9.3)	0.204
GGO, %	34.9 (32.2, 37.6)	33.6 (29.6, 37.6)	37.5 (33.3, 41.7)	0.395
Consolidation, %	9.6 (7.9, 11.4)	11.9 (9.3, 14.5)	10.6 (7.8, 13.3)	0.373
Affected parenchyma, %	44.5 (40.5, 48.5)	45.5 (39.5, 51.5)	48.1 (41.8, 54.4)	0.644
GGO-to-consolidation ratio	5.4 (5.0, 5.8)	4.2 (3.6, 4.9)	4.6 (3.9, 5.3)	**0.009** [a]

Supplementary Table S10. Results of the GLM between-group comparison, shown as adjusted means and 95% confidence intervals for the right upper lobe, middle lobe, right lower lobe, left upper lobe, and left lower lobe, as well as for the right and left lung. *a* – statistically significant difference between CTD-ILD and IPAF; *b* – statistically significant difference between CTD-ILD and IPF; *c* – statistically significant difference between IPAF and IPF; *d* – no statistically significant difference in pairwise comparison. CTD – connective tissue disease; ILD – interstitial lung disease; IPAF – interstitial

Parameter	CTD-ILD	IPAF	IPF	P-value
**Right lung**				
Total volume, ml	2194.5 (2074.6, 2314.4)	2133.0 (1953.5, 2312.6)	2198.0 (2010.9, 2385.1)	0.836
Functional parenchyma, %	66.4 (63.1, 69.8)	61.6 (56.7, 66.6)	60.9 (55.7, 66.1)	0.133
Emphysema, %	11.7 (10.1, 13.3)	11.5 (9.1, 13.9)	9.1 (6.6, 11.6)	0.215
GGO, %	27.5 (25.1, 29.8)	29.7 (26.2, 33.3)	30.6 (26.9, 34.3)	0.313
Consolidation, %	6.1 (4.8, 7.4)	8.6 (6.7, 10.5)	8.4 (6.4, 10.4)	**0.047** [d]
Affected parenchyma, %	33.6 (30.3, 36.9)	38.4 (33.4, 43.3)	39.1 (33.9, 44.3)	0.133
GGO-to-consolidation ratio	5.7 (5.4, 6.0)	4.5 (4.0, 5.0)	4.6 (4.1, 5.1)	**< 0.001** [a, b]
**Left lung**				
Total volume, ml	1857.1 (1743.8, 1970.6)	1845.5 (1675.7, 2015.3)	1882.2 (1705.3, 2059.2)	0.954
Functional parenchyma, %	65.0 (61.5, 68.5)	61.4 (56.1, 66.7)	59.9 (54.4, 65.4)	0.251
Emphysema, %	11.5 (9.9, 13.1)	11.6 (9.2, 14.0)	8.9 (6.4, 11.5)	0.209
GGO, %	28.5 (26.1, 31.0)	29.1 (25.5, 32.8)	32.0 (28.2, 35.8)	0.317
Consolidation, %	6.5 (5.2, 7.8)	9.5 (7.4, 11.5)	8.2 (6.1, 10.2)	0.051
Affected parenchyma, %	35.0 (31.5, 38.5)	38.6 (33.3, 43.9)	40.1 (34.7, 45.6)	0.251
GGO-to-consolidation ratio	5.5 (5.2, 5.8)	4.4 (3.9, 4.9)	4.8 (4.3, 5.3)	**< 0.001** [a, b]

pneumonia with autoimmune features; IPF – idiopathic pulmonary fibrosis; GGO – ground-glass opacity

Supplementary Table S10. Continued.

	Univariable Analysis		*Multivariable Analysis*	
Variable	**HR (95% CI)**	**P-value**	**HR (95% CI)**	**P-value**
Sex	0.51 (0.37, 0.71)	< 0.001	0.61 (0.41, 0.90)	0.013
Age	1.03 (1.01, 1.04)	< 0.001	1.02 (1.01, 1.04)	0.001
FEV1% predicted	0.98 (0.97, 0.99)	< 0.001	0.99 (0.97, 1.00)	0.058
FVC% predicted	0.99 (0.98, 0.99)	< 0.001	0.99 (0.98, 1.01)	0.620
Current or past smoker	1.18 (0.85, 1.64)	0.315	0.82 (0.56, 1.20)	0.306
ILD treatment	0.81 (0.58, 1.1)	0.192	0.83 (0.60, 1.17)	0.284
Total volume upper third	1.00 (0.99, 1.00)	0.380		
Percentage emphysema upper third	0.98 (0.97, 0.99)	0.039	0.98 (0.96, 1.00)	0.085
Percentage affected upper third	1.01 (1.00, 1.02)	0.018	0.99 (0.98, 1.00)	0.771
GGO-to-consolidation upper third	0.89 (0.83, 0.97)	0.005	0.93 (0.85, 1.01)	0.096
Presence of UIP pattern	2.21 (1.43, 3.40)	< 0.001	1.57 (0.99, 2.48)	0.053

Supplementary Table S11. Results of univariable and multivariable Cox regression analyses of quantitative CT data from the upper third of the lungs. HR – hazard ratio; FEV_1 – forced expiratory volume in 1 second; FVC – forced vital capacity; GGO – ground-glass opacity; UIP – usual interstitial pneumonia

	Univariable Analysis		*Multivariable Analysis*	
Variable	**HR (95% CI)**	**P-value**	**HR (95% CI)**	**P-value**
Sex	0.51 (0.37, 0.71)	< 0.001	0.63 (0.43, 0.94)	0.022
Age	1.03 (1.01, 1.04)	< 0.001	1.02 (1.01, 1.04)	0.001
FEV1% predicted	0.98 (0.97, 0.99)	< 0.001	0.99 (0.97, 0.99)	0.024
FVC% predicted	0.99 (0.98, 0.99)	< 0.001	1.00 (0.99, 1.01)	0.901
Current or past smoker	1.18 (0.85, 1.64)	0.315	0.83 (0.57, 1.21)	0.322
ILD treatment	0.81 (0.58, 1.1)	0.192	0.80 (0.57, 1.12)	0.198
Total volume middle third	1.00 (1.00, 1.00)	0.675		
Percentage emphysema middle third	0.98 (0.96, 0.99)	0.006	0.96 (0.93, 0.99)	0.005
Percentage affected middle third	1.01 (1.00, 1.02)	0.003	0.99 (0.98, 1.02)	0.229
GGO-to-consolidation middle third	0.82 (0.74, 0.92)	< 0.001	0.84 (0.74, 0.96)	0.011
Presence of UIP pattern	2.21 (1.43, 3.40)	< 0.001	1.59 (1.00, 2.50)	0.048

Supplementary Table S12. Results of univariable and multivariable Cox regression analyses of quantitative CT data from the middle third of the lungs. HR – hazard ratio; FEV_1 – forced expiratory volume in 1 second; FVC – forced vital capacity; GGO – ground-glass opacity; UIP – usual interstitial pneumonia

	Univariable Analysis		*Multivariable Analysis*	
Variable	**HR (95% CI)**	**P-value**	**HR (95% CI)**	**P-value**
Sex	0.51 (0.37, 0.71)	< 0.001	0.64 (0.43, 0.95)	0.025
Age	1.03 (1.01, 1.04)	< 0.001	1.02 (1.01, 1.04)	0.001
FEV1% predicted	0.98 (0.97, 0.99)	< 0.001	0.98 (0.97, 0.99)	0.019
FVC% predicted	0.99 (0.98, 0.99)	< 0.001	1.00 (0.99, 1.01)	0.841
Current or past smoker	1.18 (0.85, 1.64)	0.315	0.90 (0.62, 1.32)	0.601
ILD treatment	0.81 (0.58, 1.1)	0.192	0.81 (0.57, 1.13)	0.211
Total volume lower third	1.00 (0.99, 1.00)	0.213		
Percentage emphysema lower third	0.97 (0.95, 0.98)	0.003	0.95 (0.92, 0.99)	0.010
Percentage affected lower third	1.01 (1.00, 1.02)	0.002	0.99 (0.98, 0.99)	0.239
GGO-to-consolidation lower third	0.89 (0.83, 0.95)	< 0.001	0.93 (0.86, 1.02)	0.123
Presence of UIP pattern	2.21 (1.43, 3.40)	< 0.001	1.70 (1.07, 2.71)	0.025

Supplementary Table S13. Results of univariable and multivariable Cox regression analyses of quantitative CT data from the lower third of the lungs. HR – hazard ratio; FEV_1 – forced expiratory volume in 1 second; FVC – forced vital capacity; GGO – ground-glass opacity; UIP – usual interstitial pneumonia

	Univariable Analysis		*Multivariable Analysis*	
Variable	**HR (95% CI)**	**P-value**	**HR (95% CI)**	**P-value**
Sex	0.51 (0.37, 0.71)	< 0.001	0.64 (0.44, 0.95)	0.027
Age	1.03 (1.01, 1.04)	< 0.001	1.02 (1.01, 1.04)	0.002
FEV1% predicted	0.98 (0.97, 0.99)	< 0.001	0.99 (0.97, 0.99)	0.027
FVC% predicted	0.99 (0.98, 0.99)	< 0.001	1.00 (0.99, 1.01)	0.951
Current or past smoker	1.18 (0.85, 1.64)	0.315	0.84 (0.57, 1.22)	0.353
ILD treatment	0.81 (0.58, 1.1)	0.192	0.79 (0.56, 1.11)	0.177
Total volume right lung	1.00 (1.00, 1.00)	0.502		
Percentage emphysema right lung	0.98 (0.96, 0.99)	0.009	0.96 (0.93, 0.99)	0.005
Percentage affected right lung	1.01 (1.00, 1.02)	0.004	0.84 (0.97, 1.00)	0.150
GGO-to-consolidation right lung	0.80 (0.73, 0.89)	< 0.001	0.84 (0.74, 0.95)	0.005
Presence of UIP pattern	2.21 (1.43, 3.40)	< 0.001	1.54 (0.97, 2.4)	0.066

Supplementary Table S14. Results of univariable and multivariable Cox regression analyses of quantitative CT data from the right lung. HR – hazard ratio; FEV_1 – forced expiratory volume in 1 second; FVC – forced vital capacity; GGO – ground-glass opacity; UIP – usual interstitial pneumonia

	Univariable Analysis		*Multivariable Analysis*	
Variable	**HR (95% CI)**	**P-value**	**HR (95% CI)**	**P-value**
Sex	0.51 (0.37, 0.71)	< 0.001	0.59 (0.40, 0.89)	0.011
Age	1.03 (1.01, 1.04)	< 0.001	1.02 (1.01, 1.04)	0.002
FEV1% predicted	0.98 (0.97, 0.99)	< 0.001	0.99 (0.97, 0.99)	0.025
FVC% predicted	0.99 (0.98, 0.99)	< 0.001	1.00 (0.99, 1.01)	0.926
Current or past smoker	1.18 (0.85, 1.64)	0.315	0.87 (0.60, 1.27)	0.473
ILD treatment	0.81 (0.58, 1.1)	0.192	0.82 (0.58, 1.15)	0.242
Total volume left lung	1.00 (1.00, 1.00)	0.398		
Percentage emphysema left lung	0.97 (0.96, 0.99)	0.006	0.96 (0.94, 0.99)	0.014
Percentage affected left lung	1.01 (1.00, 1.02)	0.003	0.99 (0.98, 1.01)	0.270
GGO-to-consolidation left lung	0.83 (0.75, 0.91)	< 0.001	0.88 (0.78, 0.99)	0.032
Presence of UIP pattern	2.21 (1.43, 3.40)	< 0.001	1.58 (0.99, 2.50)	0.053

Supplementary Table S15. Results of univariable and multivariable Cox regression analyses of quantitative CT data from the left lung. HR – hazard ratio; FEV_1 – forced expiratory volume in 1 second; FVC – forced vital capacity; GGO – ground-glass opacity; UIP – usual interstitial pneumonia

	Univariable Analysis		*Multivariable Analysis*	
Variable	**HR (95% CI)**	**P-value**	**HR (95% CI)**	**P-value**
Sex	0.51 (0.37, 0.71)	< 0.001	0.59 (0.40, 0.87)	0.008
Age	1.03 (1.01, 1.04)	< 0.001	1.02 (1.01, 1.04)	0.002
FEV1% predicted	0.98 (0.97, 0.99)	< 0.001	0.99 (0.97, 0.99)	0.032
FVC% predicted	0.99 (0.98, 0.99)	< 0.001	1.00 (0.99, 1.01)	0.882
Current or past smoker	1.18 (0.85, 1.64)	0.315	0.83 (0.55, 1.18)	0.270
ILD treatment	0.81 (0.58, 1.1)	0.192	0.83 (0.59, 1.17)	0.284
Total volume ventral lung	1.00 (1.00, 1.00)	0.428		
Percentage emphysema ventral lung	0.98 (0.96, 0.99)	0.016	0.97 (0.94, 0.99)	0.012
Percentage affected ventral lung	1.01 (1.00, 1.02)	0.003	0.99 (0.98, 1.01)	0.443
GGO-to-consolidation ventral lung	0.82 (0.73, 0.91)	< 0.001	0.85 (0.75, 0.96)	0.010
Presence of UIP pattern	2.21 (1.43, 3.40)	< 0.001	1.49 (0.94, 2.38)	0.093

Supplementary Table S16. Results of univariable and multivariable Cox regression analyses of quantitative CT data from the ventral lung parts. HR – hazard ratio; FEV_1 – forced expiratory volume in 1 second; FVC – forced vital capacity; GGO – ground-glass opacity; UIP – usual interstitial pneumonia

	Univariable Analysis		*Multivariable Analysis*	
Variable	**HR (95% CI)**	**P-value**	**HR (95% CI)**	**P-value**
Sex	0.51 (0.37, 0.71)	< 0.001	0.64 (0.43, 0.94)	0.024
Age	1.03 (1.01, 1.04)	< 0.001	1.02 (1.00, 1.04)	0.003
FEV1% predicted	0.98 (0.97, 0.99)	< 0.001	0.98 (0.97, 0.99)	0.022
FVC% predicted	0.99 (0.98, 0.99)	< 0.001	1.00 (0.99, 1.01)	0.959
Current or past smoker	1.18 (0.85, 1.64)	0.315	0.88 (0.60, 1.28)	0.489
ILD treatment	0.81 (0.58, 1.1)	0.192	0.78 (0.56, 1.10)	0.162
Total volumes dorsal lung	1.00 (1.00, 1.00)	0.452		
Percentage emphysema dorsal lung	0.97 (0.95, 0.99)	0.005	0.96 (0.93, 0.99)	0.009
Percentage affected dorsal lung	1.01 (1.00, 1.02)	0.005	0.99 (0.98, 1.01)	0.179
GGO-to-consolidation dorsal lung	0.84 (0.78, 0.91)	< 0.001	0.88 (0.79, 0.98)	0.024
Presence of UIP pattern	2.21 (1.43, 3.40)	< 0.001	1.61 (1.02, 2.55)	0.041

Supplementary Table S17. Results of univariable and multivariable Cox regression analyses of quantitative CT data from the dorsal lung parts. HR – hazard ratio; FEV_1 – forced expiratory volume in 1 second; FVC – forced vital capacity; GGO – ground-glass opacity; UIP – usual interstitial pneumonia

	Univariable Analysis		*Multivariable Analysis*	
Variable	**HR (95% CI)**	**P-value**	**HR (95% CI)**	**P-value**
Sex	0.51 (0.37, 0.71)	< 0.001	0.62 (0.42, 0.92)	0.017
Age	1.03 (1.01, 1.04)	< 0.001	1.02 (1.01, 1.04)	0.001
FEV1% predicted	0.98 (0.97, 0.99)	< 0.001	0.99 (0.97, 0.99)	0.040
FVC% predicted	0.99 (0.98, 0.99)	< 0.001	0.99 (0.99, 1.01)	0.840
Current or past smoker	1.18 (0.85, 1.64)	0.315	0.83 (0.57, 1.21)	0.326
ILD treatment	0.81 (0.58, 1.1)	0.192	0.82 (0.59, 1.15)	0.258
Total volume RUL	1.00 (1.00, 1.00)	0.345		
Percentage emphysema RUL	0.98 (0.96, 0.99)	0.019	0.98 (0.95, 1.00)	0.056
Percentage affected RUL	1.01 (1.00, 1.02)	0.004	0.99 (0.98, 1.01)	0.652
GGO-to-consolidation RUL	0.94 (0.90, 0.97)	< 0.001	0.96 (0.91, 1.00)	0.059
Presence of UIP pattern	2.21 (1.43, 3.40)	< 0.001	1.57 (0.99, 2.48)	0.056

Supplementary Table S18. Results of univariable and multivariable Cox regression analyses of quantitative CT data from the right upper lobe. HR – hazard ratio; FEV_1 – forced expiratory volume in 1 second; FVC – forced vital capacity; GGO – ground-glass opacity; UIP – usual interstitial pneumonia, RUL – right upper lobe

	Univariable Analysis		*Multivariable Analysis*	
Variable	**HR (95% CI)**	**P-value**	**HR (95% CI)**	**P-value**
Sex	0.51 (0.37, 0.71)	< 0.001	0.59 (0.40, 0.89)	0.011
Age	1.03 (1.01, 1.04)	< 0.001	1.03 (1.01, 1.04)	< 0.001
FEV1% predicted	0.98 (0.97, 0.99)	< 0.001	0.99 (0.97, 1.00)	0.055
FVC% predicted	0.99 (0.98, 0.99)	< 0.001	0.99 (0.99, 1.01)	0.860
Current or past smoker	1.18 (0.85, 1.64)	0.315	0.89 (0.61, 1.29)	0.527
ILD treatment	0.81 (0.58, 1.1)	0.192	0.85 (0.60, 1.19)	0.338
Total volume ML	1.00 (0.99, 1.00)	0.478		
Percentage emphysema ML	0.98 (0.96, 0.99)	0.009	0.98 (0.96, 1.00)	0.063
Percentage affected ML	1.01 (1.00, 1.02)	0.007	0.99 (0.99, 1.01)	0.938
GGO-to-consolidation ML	0.99 (0.99, 1.01)	0.890	0.99 (0.99, 1.01)	0.721
Presence of UIP pattern	2.21 (1.43, 3.40)	< 0.001	1.8 (1.15, 2.85)	0.010

Supplementary Table S19. Results of univariable and multivariable Cox regression analyses of quantitative CT data from the middle lobe. HR – hazard ratio; FEV_1 – forced expiratory volume in 1 second; FVC – forced vital capacity; GGO – ground-glass opacity; UIP – usual interstitial pneumonia, ML – middle lobe

	Univariable Analysis		*Multivariable Analysis*	
Variable	**HR (95% CI)**	**P-value**	**HR (95% CI)**	**P-value**
Sex	0.51 (0.37, 0.71)	< 0.001	0.63 (0.42, 0.93)	0.019
Age	1.03 (1.01, 1.04)	< 0.001	1.02 (1.01, 1.04	0.004
FEV1% predicted	0.98 (0.97, 0.99)	< 0.001	0.98 (0.97, 0.99)	0.023
FVC% predicted	0.99 (0.98, 0.99)	< 0.001	1.00 (0.99, 1.01)	0.815
Current or past smoker	1.18 (0.85, 1.64)	0.315	0.89 (0.86, 1.30)	0.551
ILD treatment	0.81 (0.58, 1.1)	0.192	0.78 (0.56, 1.10)	0.162
Total volume RLL	1.00 (0.99, 1.00)	0.064		
Percentage emphysema RLL	0.97 (0.95, 0.99)	0.002	0.95 (0.92, 0.98)	0.004
Percentage affected RLL	1.01 (1.00, 1.02)	0.002	0.99 (0.98, 1.00)	0.167
GGO-to-consolidation RLL	0.92 (0.88, 0.96)	< 0.001	0.95 (0.90, 1.00)	0.051
Presence of UIP pattern	2.21 (1.43, 3.40)	< 0.001	1.73 (1.09, 2.75)	0.019

Supplementary Table S20. Results of univariable and multivariable Cox regression analyses of quantitative CT data from the right lower lobe. HR – hazard ratio; FEV_1 – forced expiratory volume in 1 second; FVC – forced vital capacity; GGO – ground-glass opacity; UIP – usual interstitial pneumonia; RLL – right lower lobe

	Univariable Analysis		*Multivariable Analysis*	
Variable	**HR (95% CI)**	**P-value**	**HR (95% CI)**	**P-value**
Sex	0.51 (0.37, 0.71)	< 0.001	0.59 (0.40, 0.87)	0.007
Age	1.03 (1.01, 1.04)	< 0.001	1.02 (1.01, 1.04)	0.002
FEV1% predicted	0.98 (0.97, 0.99)	< 0.001	0.99 (0.97, 0.99)	0.035
FVC% predicted	0.99 (0.98, 0.99)	< 0.001	1.00 (0.99, 1.01)	0.895
Current or past smoker	1.18 (0.85, 1.64)	0.315	0.84 (0.57, 1.20)	0.354
ILD treatment	0.81 (0.58, 1.1)	0.192	0.84 (0.60, 1.17)	0.298
Total volume LUL	1.00 (1.00, 1.00)	0.977		
Percentage emphysema LUL	0.98 (0.96, 0.99)	0.009	0.97 (0.95, 0.99)	0.038
Percentage affected LUL	1.01 (1.00, 1.02)	0.003	0.99 (0.99, 1.01)	0.626
GGO-to-consolidation LUL	0.86 (0.78, 0.95)	0.002	0.91 (0.82, 1.02)	0.090
Presence of UIP pattern	2.21 (1.43, 3.40)	< 0.001	1.57 (0.99, 2.49)	0.057

Supplementary Table S21. Results of univariable and multivariable Cox regression analyses of quantitative CT data from the left upper lobe. HR – hazard ratio; FEV_1 – forced expiratory volume in 1 second; FVC – forced vital capacity; GGO – ground-glass opacity; UIP – usual interstitial pneumonia; LUL – left upper lobe

	Univariable Analysis		*Multivariable Analysis*	
Variable	**HR (95% CI)**	**P-value**	**HR (95% CI)**	**P-value**
Sex	0.51 (0.37, 0.71)	< 0.001	0.62 (0.42, 0.92)	0.018
Age	1.03 (1.01, 1.04)	< 0.001	1.02 (1.01, 1.04)	0.001
FEV1% predicted	0.98 (0.97, 0.99)	< 0.001	0.98 (0.97, 0.99)	0.019
FVC% predicted	0.99 (0.98, 0.99)	< 0.001	1.00 (0.99, 1.01)	0.830
Current or past smoker	1.18 (0.85, 1.64)	0.315	0.91 (0.62, 1.33)	0.614
ILD treatment	0.81 (0.58, 1.1)	0.192	0.81 (0.58, 1.14)	0.227
Total volume LLL	1.00 (0.99, 1.0)	0.076		
Percentage emphysema LLL	0.97 (0.95, 0.99)	0.004	0.96 (0.93, 0.99)	0.023
Percentage affected LLL	1.01 (1.00, 1.02)	0.006	0.99 (0.98, 1.01)	0.457
GGO-to-consolidation LLL	0.89 (0.82, 0.96)	0.004	0.94 (0.85, 1.01)	0.173
Presence of UIP pattern	2.21 (1.43, 3.40)	< 0.001	1.68 (1.06, 2.65)	0.027

Supplementary Table S22. Results of univariable and multivariable Cox regression analyses of quantitative CT data from the left lower lobe. HR – hazard ratio; FEV_1 – forced expiratory volume in 1 second; FVC – forced vital capacity; GGO – ground-glass opacity; UIP – usual interstitial pneumonia; LLL – left lower lobe

	Univariable Analysis		***Multivariable Analysis***	
Variable	**HR (95% CI)**	**P-value**	**HR (95% CI)**	**P-value**
Sex	0.51 (0.37, 0.71)	< 0.001	0.59 (0.40, 0.88)	0.009
Age	1.03 (1.01, 1.04)	< 0.001	1.03 (1.01, 1.04)	< 0.001
FEV1% predicted	0.98 (0.97, 0.99)	< 0.001	0.99 (0.97, 1.00)	0.047
FVC% predicted	0.99 (0.98, 0.99)	< 0.001	1.00 (0.98, 1.01)	0.605
Current or past smoker	1.18 (0.85, 1.64)	0.315	0.85 (0.58, 1.23)	0.388
ILD treatment	0.81 (0.58, 1.1)	0.192	0.88 (0.63, 1.23)	0.466
Presence of UIP pattern	2.21 (1.43, 3.40)	< 0.001	1.64 (1.05, 2.57)	0.029
GGO (%)	1.01 (1.00, 1.02)	0.044	1.00 (0.99, 1.02)	0.524

Supplementary Table S23. Results of an alternative model (Model A) including whole-lung GGO percentage as the quantitative CT parameter in univariable and multivariable Cox regression analyses. HR = hazard ratio; FEV_1 = forced expiratory volume in 1 second; FVC = forced vital capacity; GGO = ground-glass opacity; UIP = usual interstitial pneumonia

	Univariable Analysis		***Multivariable Analysis***	
Variable	**HR (95% CI)**	**P-value**	**HR (95% CI)**	**P-value**
Sex	0.51 (0.37, 0.71)	< 0.001	0.59 (0.40, 0.86)	0.007
Age	1.03 (1.01, 1.04)	< 0.001	1.02 (1.01, 1.04)	0.001
FEV1% predicted	0.98 (0.97, 0.99)	< 0.001	0.99 (0.97, 1.00)	0.038
FVC% predicted	0.99 (0.98, 0.99)	< 0.001	1.00 (0.99, 1.01)	0.984
Current or past smoker	1.18 (0.85, 1.64)	0.315	0.81 (0.56, 1.18)	0.275
ILD treatment	0.81 (0.58, 1.1)	0.192	0.85 (0.61, 1.18)	0.323
Presence of UIP pattern	2.21 (1.43, 3.40)	< 0.001	1.63 (1.05, 2.54)	0.031
Consolidation (%)	1.06 (1.03, 1.08)	< 0.001	1.04 (1.02, 1.07)	0.002

Supplementary Table S24. Results of an alternative model (Model B) including whole-lung Consolidation percentage as the quantitative CT parameter in univariable and multivariable Cox regression analyses. HR = hazard ratio; FEV_1 = forced expiratory volume in 1 second; FVC = forced vital capacity; GGO = ground-glass opacity; UIP = usual interstitial pneumonia

	Univariable Analysis		***Multivariable Analysis***	
Variable	**HR (95% CI)**	**P-value**	**HR (95% CI)**	**P-value**
Sex	0.51 (0.37, 0.71)	< 0.001	0.59 (0.40, 0.88)	0.007
Age	1.03 (1.01, 1.04)	< 0.001	1.03 (1.01, 1.04)	0.001
FEV1% predicted	0.98 (0.97, 0.99)	< 0.001	0.99 (0.97, 1.00)	0.049
FVC% predicted	0.99 (0.98, 0.99)	< 0.001	0.99 (0.98, 1.01)	0.793
Current or past smoker	1.18 (0.85, 1.64)	0.315	0.85 (0.58, 1.23)	0.377
ILD treatment	0.81 (0.58, 1.1)	0.192	0.87 (0.62, 1.21)	0.411
Presence of UIP pattern	2.21 (1.43, 3.40)	< 0.001	1.66 (1.06, 2.59)	0.026
Affected lung parenchyma (%)	1.01 (1.00, 1.02)	0.003	1.00 (0.99, 1.02)	0.149

Supplementary Table S25. Results of an alternative model (Model C) including affected lung parenchyma percentage as the quantitative CT parameter in univariable and multivariable Cox regression analyses. HR = hazard ratio; FEV_1 = forced expiratory volume in 1 second; FVC = forced vital capacity; GGO = ground-glass opacity; UIP = usual interstitial pneumonia

	Univariable Analysis		Multivariable Analysis	
Variable	**HR (95% CI)**	**P-value**	**HR (95% CI)**	**P-value**
Sex	0.51 (0.37, 0.71)	< 0.001	0.61 (0.42, 0.9)	0.013
Age	1.03 (1.01, 1.04)	< 0.001	1.02 (1.01, 1.04)	0.001
FEV1% predicted	0.98 (0.97, 0.99)	< 0.001	0.99 (0.97, 0.99)	0.026
FVC% predicted	0.99 (0.98, 0.99)	< 0.001	1.00 (0.98, 1.01)	0.742
Current or past smoker	1.18 (0.85, 1.64)	0.315	0.78 (0.54, 1.14)	0.201
ILD treatment	0.81 (0.58, 1.1)	0.192	0.85 (0.61, 1.20)	0.352
Presence of UIP pattern	2.21 (1.43, 3.40)	< 0.001	1.57 (1.01, 2.45)	0.047
GGO (%)	1.01 (1.00, 1.02)	0.044	0.98 (0.97, 1.00)	0.082
Consolidation (%)	1.06 (1.03, 1.08)	< 0.001	1.07 (1.03, 1.10)	< 0.001

Supplementary Table S26. Results of an alternative model (Model D) including whole-lung GGO and consolidation percentage as the quantitative CT parameter in univariable and multivariable Cox regression analyses. HR = hazard ratio; FEV_1 = forced expiratory volume in 1 second; FVC = forced vital capacity; GGO = ground-glass opacity; UIP = usual interstitial pneumonia

	Univariable Analysis		Multivariable Analysis	
Variable	**HR (95% CI)**	**P-value**	**HR (95% CI)**	**P-value**
Sex	0.51 (0.37, 0.71)	< 0.001	0.62 (0.42, 0.91)	0.016
Age	1.03 (1.01, 1.04)	< 0.001	1.02 (1.01, 1.04)	0.003
FEV1% predicted	0.98 (0.97, 0.99)	< 0.001	0.99 (0.97, 0.99)	0.027
FVC% predicted	0.99 (0.98, 0.99)	< 0.001	0.99 (0.98, 1.01)	0.670
Current or past smoker	1.18 (0.85, 1.64)	0.315	0.81 (0.56, 1.18)	0.275
ILD treatment	0.81 (0.58, 1.1)	0.192	0.87 (0.63, 1.21)	0.416
Presence of UIP pattern	2.21 (1.43, 3.40)	< 0.001	1.45 (1.92, 2.28)	0.109
GGO-to-consolidation ratio	0.84 (0.72, 0.89)	< 0.001	0.89 (0.80, 0.99)	0.038

Supplementary Table S27. Results of an alternative model (Model E) including whole-lung GGO-to-Consolidation ratio as the quantitative CT parameter in univariable and multivariable Cox regression analyses. HR = hazard ratio; FEV_1 = forced expiratory volume in 1 second; FVC = forced vital capacity; GGO = ground-glass opacity; UIP = usual interstitial pneumonia

Model	*QCT Variable*	*HR (95% CI)*	*p-value*	*-2 Log Likelihood*
A	GGO (%)	1.00 (0.99–1.02)	0.524	1324.02
B	Affected lung (%)	1.01 (0.99–1.02)	0.149	1322.39
C	Consolidation (%)	1.04 (1.02–1.07)	0.002	1316.36
D	Consolidation	1.07 (1.03–1.10)	< 0.001	1313.29
	GGO	0.98 (0.97–1.00)	0.082	
E	GGO-to-consolidation ratio	0.89 (0.80–0.99)	0.038	1319.94

Supplementary Table S28. Comparison of key metrics from alternative multivariable Cox regression models (A – E), including hazard ratios, 95% confidence intervals, p values, and −2 log likelihood. HR – hazard ratio; GGO – ground-glass opacity

Model	*Variable*	***CTD-ILD***		***IPAF***		***IPF***	
	Variable	**HR (95% CI)**	**P-value**	**HR (95% CI)**	**P-value**	**HR (95% CI)**	**P-value**
1	*Age*	1.01 (0.99, 1.03)	0.267	1.04 (1.00, 1.14)	0.047	1.02 (0.98, 1.06)	0.384
	Sex	0.55 (0.33, 0.92)	0.023	0.96 (0.47, 1.96)	0.906	1.41 (0.66, 2.99)	0.377
	GGO-to-consolidation whole lung	0.84 (0.73, 0.98)	0.025	0.91 (0.73, 1.14)	0.419	0.91 (0.72, 1.16)	0.446
2	*Age*	1.01 (0.99, 1.03)	0.160	1.04 (1.01, 1.08)	0.021	1.02 (0.98, 1.05)	0.382
	Sex	0.53 (0.32, 0.88)	0.014	0.99 (0.49, 2.03)	0.986	1.58 (0.71, 3.52)	0.258
	GGO-to-consolidation upper third	0.93 (0.82, 1.06)	0.278	1.06 (0.88, 1.27)	0.546	0.91 (0.76, 1.08)	0.281
3	*Age*	1.01 (0.99, 1.03)	0.201	1.04 (1.00, 1.08)	0.028	1.02 (0.98, 1.06)	0.358
	Sex	0.54 (0.33, 0.91)	0.020	0.99 (0.49, 2.02)	0.978	1.45 (0.66, 3.17)	0.352
	GGO-to-consolidation middle third	0.85 (0.72, 1.01)	0.062	0.98 (0.77, 1.25)	0.896	0.93 (0.73, 1.18)	0.542
4	*Age*	1.01 (0.99, 1.03)	0.160	1.03 (0.99, 1.07)	0.069	1.02 (0.98, 1.06)	0.347
	Sex	0.52 (0.31, 0.87)	0.013	0.92 (0.45, 1.89)	0.819	1.33 (0.63, 2.82)	0.453

GGO-to-consolidation lower third	0.94 (0.85, 1.03)	0.169	0.91 (0.78, 1.08)	0.280	0.96 (0.81, 1.15)	0.675

Supplementary Table S29. Results of the exploratory multivariable Cox regression analysis of the GGO-to-consolidation ratio for each lung compartment and ILD subgroup. HR – hazard ratio; CI – confidence interval; CTD – connective tissue disease; ILD – interstitial lung disease; IPAF – interstitial pneumonia with autoimmune features; IPF – idiopathic pulmonary fibrosis; GGO – ground-glass opacity; RUL – right upper lobe; ML – middle lobe; RLL – right lower lobe; LUL – left upper lobe; LLL – left lower lobe

Model	*Variable*	***CTD-ILD***		***IPAF***		***IPF***	
	Variable	**HR (95% CI)**	**P-value**	**HR (95% CI)**	**P-value**	**HR (95% CI)**	**P-value**
5	*Age*	1.01 (0.99, 1.03)	0.306	1.04 (1.00, 1.07)	0.037	1.02 (0.98, 1.06)	0.368
	Sex	0.56 (0.33, 0.94)	0.027	0.98 (0.48, 2.00)	0.950	1.40 (0.66, 2.99)	0.383
	GGO-to-consolidation right lung	0.84 (0.73, 0.97)	0.019	0.94 (0.75, 1.18)	0.594	0.93 (0.74, 1.16)	0.519
6	*Age*	1.01 (0.99, 1.03)	0.189	1.04 (0.99, 1.07	0.058	1.02 (0.98, 1.06)	0.398
	Sex	0.54 (0.32, 0.90)	0.017	0.93 (0.46, 1.91)	0.850	1.39 (0.66, 2.95)	0.389
	GGO-to-consolidation left lung	0.87 (0.75, 1.01)	0.064	0.89 (0.72, 1.10)	0.282	0.91 (0.72, 1.15)	0.425
7	*Age*	1.01 (0.99, 1.03)	0.226	1.04 (1.00, 1.08)	0.028	1.02 (0.98, 1.06)	0.376
	Sex	0.55 (0.33, 0.92)	0.226	0.99 (0.49, 2.03)	0.980	1.37 (0.65, 2.89)	0.414

	GGO-to-consolidation ventral lung	0.87 (0.74, 1.02)	0.083	0.99 (0.79, 1.26)	0.998	0.90 (0.73, 1.12)	0.335
	Age	1.01 (0.99, 1.03)	0.224	1.04 (0.99, 1.07)	0.057	1.02 (0.98, 1.06)	0.363
8	*Sex*	0.54 (0.32, 0.90)	0.019	0.94 (0.46, 1.93)	0.867	1.39 (0.65, 2.96)	0.398
	GGO-to-consolidation dorsal lung	0.88 (0.77, 0.99)	0.039	0.89 (0.72, 1.10)	0.291	0.95 (0.76, 1.18)	0.650

Supplementary Table S29. Continued.

Model	*Variable*	***CTD-ILD***		***IPAF***		***IPF***	
	Variable	**HR (95% CI)**	**P-value**	**HR (95% CI)**	**P-value**	**HR (95% CI)**	**P-value**
	Age	1.01 (0.99, 1.03)	0.190	1.04 (1.00, 1.07)	0.023	1.02 (0.98, 1.06)	0.380
9	*Sex*	0.56 (0.33, 0.94)	0.028	0.99 (0.49, 2.03)	0.989	1.37 (0.65, 2.91)	0.411
	GGO-to-consolidation RUL	0.96 (0.89, 1.02)	0.194	1.01 (0.94, 1.10)	0.697	0.97 (0.90, 1.05)	0.444
	Age	1.02 (0.99, 1.03)	0.074	1.04 (1.00, 1.07)	0.028	1.02 (0.98, 1.05)	0.340
10	*Sex*	0.52 (0.31, 0.86)	0.012	0.90 (0.43, 1.88)	0.772	1.78 (0.80, 3.98)	0.159
	GGO-to-consolidation ML	0.99 (0.89, 1.10)	0.808	1.09 (0.82, 1.45)	0.558	0.96 (0.99, 1.00)	0.222
	Age	1.01 (0.99, 1.03)	0.249	1.04 (0.99, 1.07)	0.054	1.02 (0.98, 1.06)	0.364
11	*Sex*	0.53 (0.32, 0.88)	0.014	0.95 (0.47, 1.95)	0.892	1.35 (0.64, 2.87)	0.426

	GGO-to-consolidation RLL	0.94 (0.88, 1.00)	0.057	0.94 (0.84, 1.05)	0.247	0.98 (0.88, 1.09)	0.709

Supplementary Table S29. Continued.

Model	*Variable*	***CTD-ILD***		***IPAF***		***IPF***	
	Variable	**HR (95% CI)**	**P-value**	**HR (95% CI)**	**P-value**	**HR (95% CI)**	**P-value**
12	*Age*	1.01 (0.99, 1.03)	0.176	1.04 (1.00, 1.08)	0.030	1.02 (0.98, 1.05)	0.408
	Sex	0.53 (0.32, 0.88)	0.014	0.98 (0.48, 2.01)	0.960	1.41 (0.66, 3.01)	0.372
	GGO-to-consolidation LUL	0.93 (0.79, 1.08)	0.348	0.98 (0.79, 1.21)	0.831	0.91 (0.73, 1.13)	0.380
13	*Age*	1.02 (0.99, 1.03)	0.092	1.03 (0.99, 1.07)	0.086	1.02 (0.98, 1.06)	0.359
	Sex	0.52 (0.31, 0.86)	0.011	0.91 (0.44, 1.86)	0.794	1.35 (0.64, 2.84)	0.435
	GGO-to-consolidation LLL	0.95 (0.85, 1.06)	0.348	0.87 (0.72, 1.06)	0.175	0.95 (0.78, 1.16)	0.607

Supplementary Table S29. Continued.
